# Cooperation between Different CRISPR-Cas Types Enables Adaptation in an RNA-Targeting System

**DOI:** 10.1128/mBio.03338-20

**Published:** 2021-03-30

**Authors:** Ville Hoikkala, Janne Ravantti, César Díez-Villaseñor, Marja Tiirola, Rachel A. Conrad, Mark J. McBride, Sylvain Moineau, Lotta-Riina Sundberg

**Affiliations:** a University of Jyväskylä Department of Biological and Environmental Science, Nanoscience Center, Jyväskylä, Finland; b University of Helsinki, Molecular and Integrative Biosciences Research Programme, Helsinki, Finland; c University of Wisconsin—Milwaukee, Department of Biological Sciences, Milwaukee, Wisconsin, USA; d Université Laval, Département de Biochimie, de Microbiologie, et de Bio-informatique, Québec City, Québec, Canada; University of Otago; Department of Veterinary Medicine, University of Cambridge

**Keywords:** CRISPR, adaptation, bacteriophages, coevolution, spacer acquisition, type II, type VI

## Abstract

CRISPR-Cas immune systems adapt to new threats by acquiring new spacers from invading nucleic acids such as phage genomes. However, some CRISPR-Cas loci lack genes necessary for spacer acquisition despite variation in spacer content between microbial strains. It has been suggested that such loci may use acquisition machinery from cooccurring CRISPR-Cas systems within the same strain. Here, following infection by a virulent phage with a double-stranded DNA (dsDNA) genome, we observed spacer acquisition in the native host Flavobacterium columnare that carries an acquisition-deficient CRISPR-Cas subtype VI-B system and a complete subtype II-C system. We show that the VI-B locus acquires spacers from both the bacterial and phage genomes, while the newly acquired II-C spacers mainly target the viral genome. Both loci preferably target the terminal end of the phage genome, with priming-like patterns around a preexisting II-C protospacer. Through gene deletion, we show that the RNA-cleaving VI-B system acquires spacers in *trans* using acquisition machinery from the DNA-cleaving II-C system. Our observations support the concept of cross talk between CRISPR-Cas systems and raise further questions regarding the plasticity of adaptation modules.

## Abstract (precis)

CRISPR-Cas systems are immune systems that protect bacteria and archaea against their viruses, bacteriophages. Immunity is achieved through the acquisition of short DNA fragments from the viral invader’s genome.

## INTRODUCTION

CRISPR (clustered regularly interspaced short palindromic repeat) arrays consist of multiple nucleotide repeats separated by variable spacer sequences (1). The repeats and spacers, together with Cas (CRISPR-associated) genes/proteins (2), constitute the CRISPR-Cas systems that protect bacteria and archaea against infections by bacteriophages (phages) (3). CRISPR-Cas systems are divided into six types and several subtypes on the basis of their *cas* gene composition (4). Immunity operates through three main phases: adaptation, expression, and interference. In the adaptation (or acquisition) phase, a fragment from the phage genome (protospacer [5]), usually next to a protospacer-adjacent motif (PAM) (6), is inserted into a CRISPR array as a spacer. New spacers are usually inserted into the array in a polarized fashion so that one end of the array (called the variable end) accumulates the most recent spacers. The variable CRISPR end is adjacent to the AT-rich region called the leader sequence (2, 3, 5, 7). In the expression phase, the array is transcribed (usually initiated from a promoter in the leader sequence and progressing toward the array) and processed to produce short CRISPR RNAs (crRNAs). These RNA fragments, each consisting of a complete or partial single spacer and repeat, guide endonucleases to complementary sequences on the invading phage genome in the interference phase (8–10). Most types of CRISPR-Cas systems target DNA, but type VI systems target RNA, while type III systems target both nucleic acids (4). Upon recognizing the PAM sequence (or the shorter protospacer-flanking site [PFS] in type VI systems [11]) and binding to the target, the target is cleaved, curing the host of infection.

Adaptation is almost universally mediated by a complex consisting of Cas1 and Cas2 that may recruit additional proteins depending on the subtype. For example, type I-D requires Cas4 for correct adaptation (12), while type II-A recruits Csn2 and Cas9 for successful recognition of the spacer acquisition motif (SAM) (13–15). The SAM is sometimes used to denote the PAM during acquisition, as it may differ from the interference-related PAM, also called the target interference motif (TIM) (13). Some subtype III-B systems acquire spacers from RNA using a Cas1 fused to a reverse transcriptase (16), which has also been predicted for some type VI-A systems (17).

Spacer acquisition generally occurs through either naive or primed adaptation. Naive adaptation occurs when no preexisting spacers target the incoming genome (18), while primed adaptation benefits from partially or fully matching preexisting spacers (19–22). Priming has been generally studied in class I systems, but recent analyses suggest that priming may be widespread in type II systems (23) and has experimentally been shown in subtype II-A (24). Type II-A priming relies on Cas9-mediated cleavage through a perfectly matching spacer and its target, leading to the production of free DNA ends that are used as material for spacer acquisition (24, 25).

Regardless of the composition of the adaptation complex, the insertion of new spacers requires interaction between the acquisition complex and the CRISPR array’s leader-repeat junction (18). This interaction relies on conserved sequences in the leaders and repeats. In the subtype II-A system of Streptococcus thermophilus, the conservation of the first 10 nucleotides on both sides of the leader-repeat junction is essential for spacer integration (26), as is the conservation of the first 41 to 43 nucleotides of the leader in the Escherichia coli subtype I-E system (18, 27). Subtype I-D leaders also contain conserved nucleotides that are more distant from the leader-repeat junction (28). As repeats and leaders vary in sequence and length, their respective adaptation proteins have coevolved accordingly (13, 29–32). Therefore, CRISPR-Cas systems cooccurring within a genome have distinct versions of Cas1 and Cas2 (and possibly other acquisition-related proteins), restraining their capacity to function on other leaders and repeats. Surprisingly, some bacterial isolates contain CRISPR-Cas systems that lack adaptation modules but still have variable spacer contents (13, 33–36). Such systems may engage in cross talk between other intragenomic CRISPR-Cas loci by utilizing their adaptation machinery in *trans* (33, 37–41). In Sulfolobus solfataricus, in *trans* acquisition is supported by an acquisition-deficient subtype III-B locus that can still acquire spacers with PAMs matching those of a cooccurring type I system with similar CRISPR sequences (35). Other examples are acquisition-deficient plasmid-encoded subtype IV-A3 loci that frequently cooccur with host-encoded subtype I-E systems and share similarities with their leader, repeat, and PAM sequences (36). Cross talk between different CRISPR-Cas systems has been shown in the expression (42) and interference (43) phases, but the evidence for in *trans*-mediated spacer acquisition between CRISPR-Cas systems is still indirect, awaiting experimental verification.

The recently discovered type VI CRISPR-Cas systems (11, 37, 39) often lack Cas1/2 (4) and thus have been proposed to acquire spacers in *trans* (37, 39–41). However, as type VI loci exclusively target RNA, obtaining spacers from double-stranded DNA (dsDNA) (the *modus operandi* for most acquisition complexes) is not optimal, as only half of the potential spacers may functionally target mRNAs. Adaptation in type VI systems has yet to be experimentally demonstrated, and it is unknown if new spacers are acquired through the in *trans* adaptation model and, if so, how this affects their capacity to target RNA.

**FIG 1:**
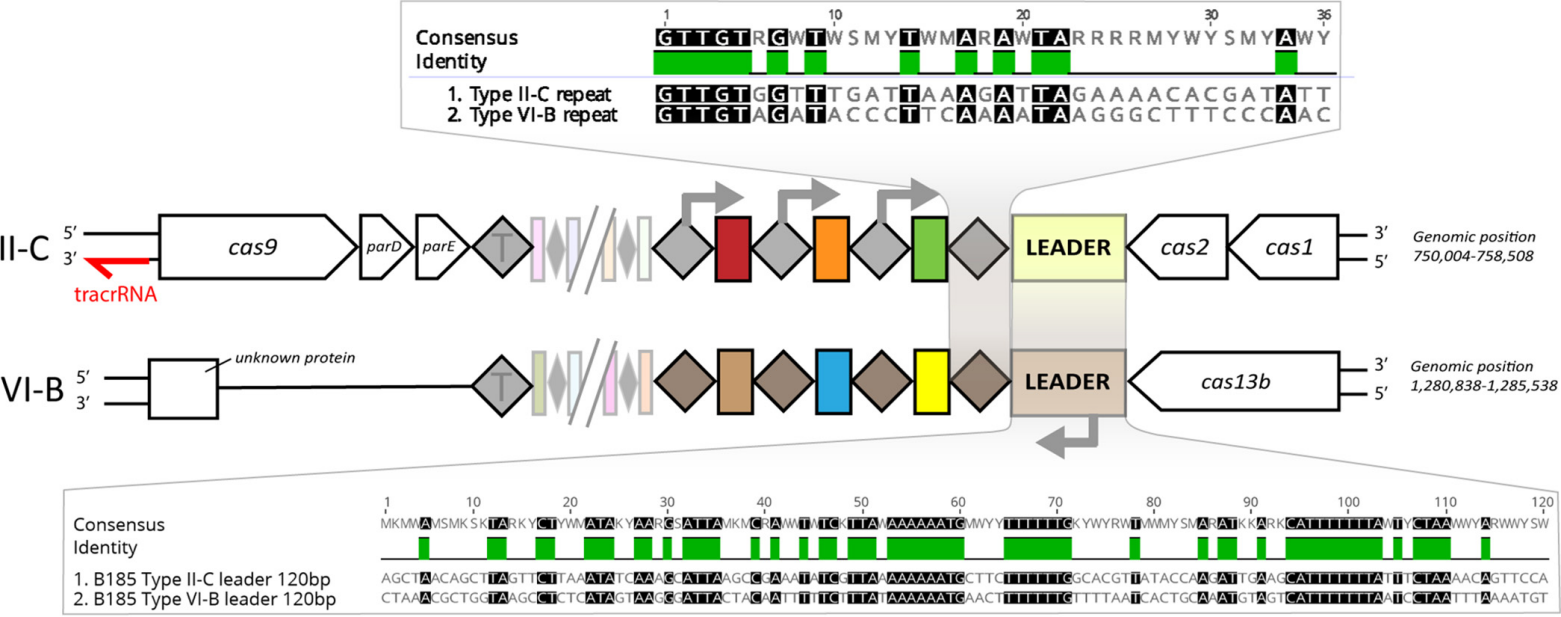
Comparison of the arrangements of II-C and VI-B CRISPR-Cas loci in *F. columnare* B185. Colored boxes represent spacers, and diamonds represent repeats (T denotes the terminal repeat). Repeats and leaders are aligned with no gaps and presented in the 5′-to-3′ direction (base 36 in repeats is leader proximal). Genomic positions are given according to the *F. columnare* B185 genome (GenBank accession no. NZ_CP010992.1). The gray arrows show the directions of transcription that are inferred from previous studies, the presence of putative promoters, and our transcriptome data. The II-C locus also contains the predicted toxin-antitoxin genes *parD* and *parE*. To make comparisons of the loci easier, both loci are displayed with the leader in the 3′ end. The putative II-C *trans*-activating crRNA (tracrRNA) has not been experimentally validated.

The genome of the fish pathogen Flavobacterium columnare has subtype II-C and VI-B CRISPR-Cas loci (Fig. 1). The II-C locus contains the adaptation proteins Cas1 and Cas2 and the endonuclease Cas9 that cleaves dsDNA (although some Cas9 variants also cleave single-stranded RNA [ssRNA] [44, 45]). The II-C locus of F. columnare also contains genes encoding a predicted ParDE type II toxin-antitoxin system between *cas9* and the repeat-spacer array, which may contribute to the immune response (46, 47). II-C systems differ from other CRISPR-Cas systems by the expression of their array from repeat-encoded promoters instead of the leader sequence (44, 48). Subtype VI-B of *F. columnare* encodes only Cas13b and does not contain adaptation genes, nor does it contain *csx27* or *csx28* that is often associated with type VI-B systems (4). Cas13 exclusively cleaves RNA and is guided to its target by its bound crRNA, similar to other interference complexes. However, once activated by this primary target, Cas13 becomes a promiscuous RNase that also cleaves noncomplementary phage and host transcripts, potentially leading to cellular dormancy or death (11, 39, 49).

**FIG 2:**
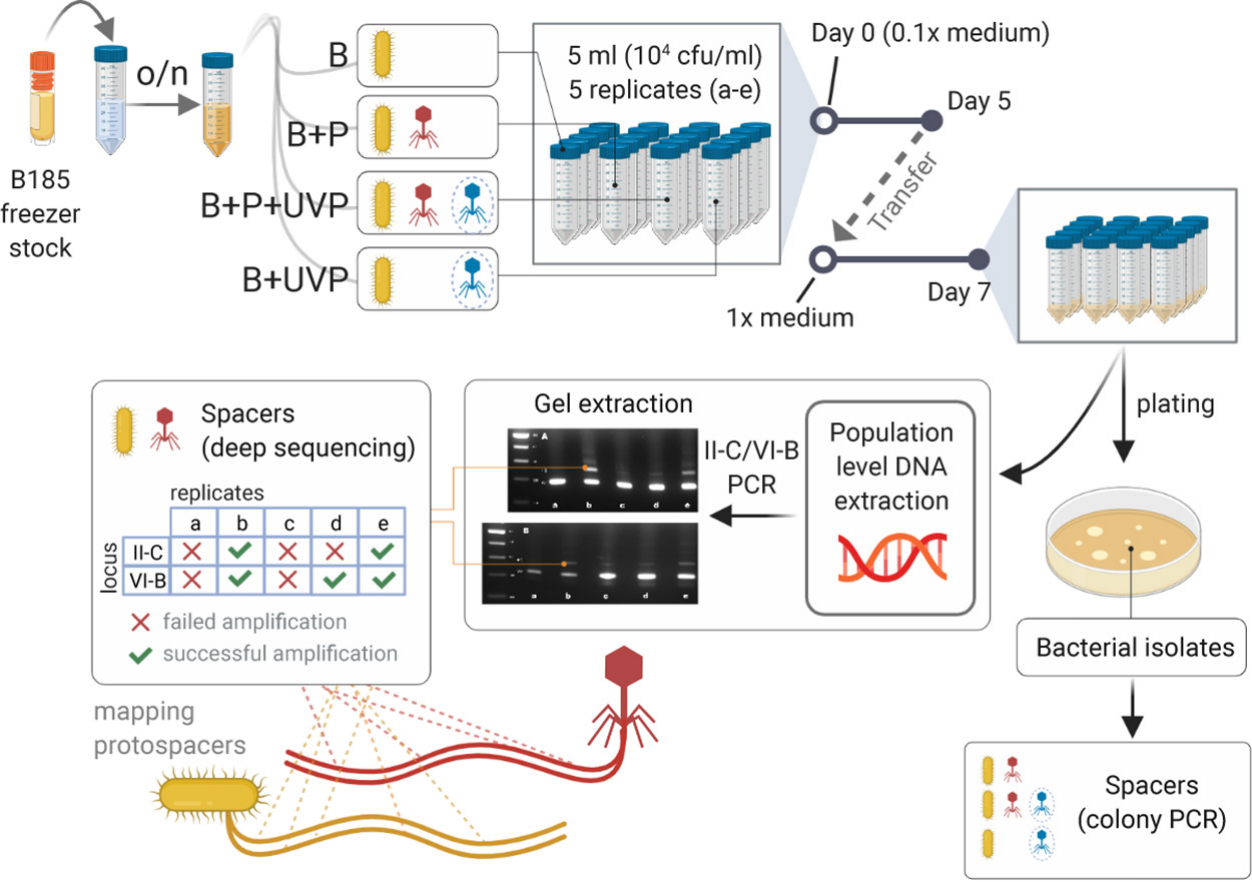
Overview of the acquisition experiment. Spacer acquisition in *F. columnare* strain B185 (B) was investigated using a two-step growth method in diluted and undiluted media. Bacterium plus phage FCL-2 (B+P) treatment was accompanied by a phage mixture containing UV-treated phages (B+P+UVP) and UV-treated phages alone (B+UVP), all done in 5 replicates (a to e). Spacers were examined in individual colonies from all three phage-based treatments as well as by deep sequencing of the CRISPR arrays in the B+P treatment. “Failed amplification” refers to samples where the DNA yield from expanded arrays was insufficient for sequencing.

Previously, we showed that natural isolates of *F. columnare* vary in their phage-targeting spacer contents in both loci, driving the evolution of sympatric phages (34). Only the phage mRNA strand was targeted by VI-B spacers (*n* = 15), while this was the case for only half of the II-C protospacers (34). While the accumulation of mRNA-targeting spacers in the VI-B locus was clear, it is unknown if this was due to a selective spacer acquisition process or from positive selection due to successful interference. To further characterize spacer acquisition in these loci, we cocultured *F. columnare* with a virulent dsDNA phage under laboratory conditions (Fig. 2). Our results revealed details of subtype VI-B and II-C adaptation in a native host as well as the first experimental evidence of in *trans* adaptation between CRISPR-Cas types despite differences in leader and repeat sequences.

## RESULTS

### Phage-induced acquisition in both loci.

To study the adaptation phase of subtype II-C and VI-B loci in a controlled environment, we cultured *F. columnare* strain B185 (50, 51) (GenBank accession no. NZ_CP010992.1) with its virulent dsDNA phage FCL-2 (accession no. NC_027125.1) (51) in liquid medium (bacterium plus phage [B+P] treatment) (Fig. 2). We also included treatments with UV-irradiated phage FCL-2 (bacteria plus UV-phage [B+UVP]) and a mixture of irradiated and nonirradiated phages (B+P+UVP). The addition of defective UV-treated phages has been shown to increase spacer acquisition in subtype II-A (52). The native II-C CRISPR array of *F. columnare* B185 contains four preexisting spacers targeting the genome of phage FCL-2 and four VI-B spacers with imperfect matches to FCL-2 (see Table S3A and B in the supplemental material). After a week of coculturing, we plated the liquid cultures to screen for expanded CRISPR arrays in individual colonies (total of 272 colonies screened) (Table S1). Only bacteria that were exposed to the phage mixture (B+P+UVP) had acquired new spacers (14 out of 85 colonies). We did not observe spacer acquisition in bacteria that were not exposed to phage (B) or that were exposed to either phage treatment alone (B+P or B+UVP) (Tables S1 and S2).

10.1128/mBio.03338-20.7 TABLE S1 (A) Results from colony screening. “Mutants” refers to isolates with an expanded II-C or VI-B array. ^A^, either bacteria alone (B), bacteria plus phages (B+P), bacteria plus UV-treated phages (B+UVP), or bacteria plus phages and UV-treated phages (B+P+UVP). ^B^, number of mutants found after the first plating on agar. ^C^, number of colonies after multiple rounds of serially plating the initial colony. (B) Colonies with additional spacers and their sequences obtained after serial plating. ORF numbers indicate phage genes, and “self” indicates bacterial chromosomal genes. Download Table S1, DOCX file, 0.02 MB. Copyright © 2021 Hoikkala et al. 2021 Hoikkala et al. https://creativecommons.org/licenses/by/4.0/ This content is distributed under the terms of the Creative Commons Attribution 4.0 International license.

10.1128/mBio.03338-20.8 TABLE S2 (A) Primers used in the study. (B) Ion Torrent reads and spacer counts from different samples. B+P refers to the treatment with bacteria plus phages. Download Table S2, DOCX file, 0.01 MB. Copyright © 2021 Hoikkala et al. 2021 Hoikkala et al. https://creativecommons.org/licenses/by/4.0/ This content is distributed under the terms of the Creative Commons Attribution 4.0 International license.

10.1128/mBio.03338-20.9 TABLE S3 (A) Preexisting type II-C spacers in *F. columnare* strain B185. Array positions indicate distances from the variable end (with 1 being the most recently acquired spacer). Target strand indicates the coding (plus [+]) or template (minus [−]) strand: any crRNA complementary to predicted ORF mRNA is targeting the plus strand. Underlined sequences in the crRNA column and * in the target column indicate mismatches in the corresponding protospacer regions. (B) Preexisting type VI-B spacers in *F. columnare* strain B185. Array positions indicate distances from the variable end (with 1 being the most recently acquired spacer). Target strand indicates the coding (plus) or template (minus) strand: any crRNA complementary to predicted ORF mRNA is targeting the plus strand. (C) Preexisting type II-C spacers in *F. columnare* strain B245. Array positions indicate distances from the variable end (with 1 being the most recently acquired spacer). Target strand indicates the coding (plus) or template (minus) strand: any crRNA complementary to predicted ORF mRNA is targeting the plus strand. Underlined sequences in the crRNA column and * in the target column indicate mismatches in the corresponding protospacer regions. (D) Preexisting type VI-B spacers in *F. columnare* strain B245. Array positions indicate distances from the variable end (with 1 being the most recently acquired spacer). Target strand indicates the coding (plus) or template (minus) strand: any crRNA complementary to predicted ORF mRNA is targeting the plus strand. Underlined sequences in the crRNA column and * in the target column indicate mismatches in the corresponding protospacer regions. Download Table S3, DOCX file, 0.03 MB. Copyright © 2021 Hoikkala et al. 2021 Hoikkala et al. https://creativecommons.org/licenses/by/4.0/ This content is distributed under the terms of the Creative Commons Attribution 4.0 International license.

Next, we focused on CRISPR adaptation at the bacterial population level by amplifying by PCR the variable ends of both arrays directly from the same liquid cultures. In contrast to individual colony screening, we observed spacer acquisition in both the phage mixture treatment (B+P+UVP) (data not shown) and the phage-only treatment (B+P) (Fig. S1). The difference between the individual-colony screening and population-level screening likely stems from the higher sensitivity of the latter. All downstream analyses were done on the B+P samples, as this treatment is closer to a natural scenario (UV irradiation lowers phage infectivity by 2 orders of magnitude). Within each replicate of the B+P treatment (identified as replicates a to e), the relative rates of spacer acquisition were similar between the two arrays (Fig. S1). To identify the diversity and origin of the new CRISPR spacers, we deep sequenced the population-scale variable ends of the CRISPR arrays. We obtained data on new II-C spacers in replicates b and e and on VI-B spacers in replicates b, d, and e (other replicates did not allow for deep sequencing due to low DNA yields). PCR amplicons in the wild-type control cultures were also sequenced to ensure that the arrays had remained unchanged.

10.1128/mBio.03338-20.1 FIG S1 Gel electrophoresis of type II-C and VI-B CRISPR arrays in Flavobacterium columnare B185. The variable ends of the arrays were amplified from population-level DNA samples of five replicate cultures (a to e) in the presence or absence of phage FCL-2. (A) II-C array with phages; (B) VI-B array with phages; (C) II-C array without phages; (D) VI-B array without phages. Download FIG S1, TIF file, 1.8 MB. Copyright © 2021 Hoikkala et al. 2021 Hoikkala et al. https://creativecommons.org/licenses/by/4.0/ This content is distributed under the terms of the Creative Commons Attribution 4.0 International license.

**FIG 3:**
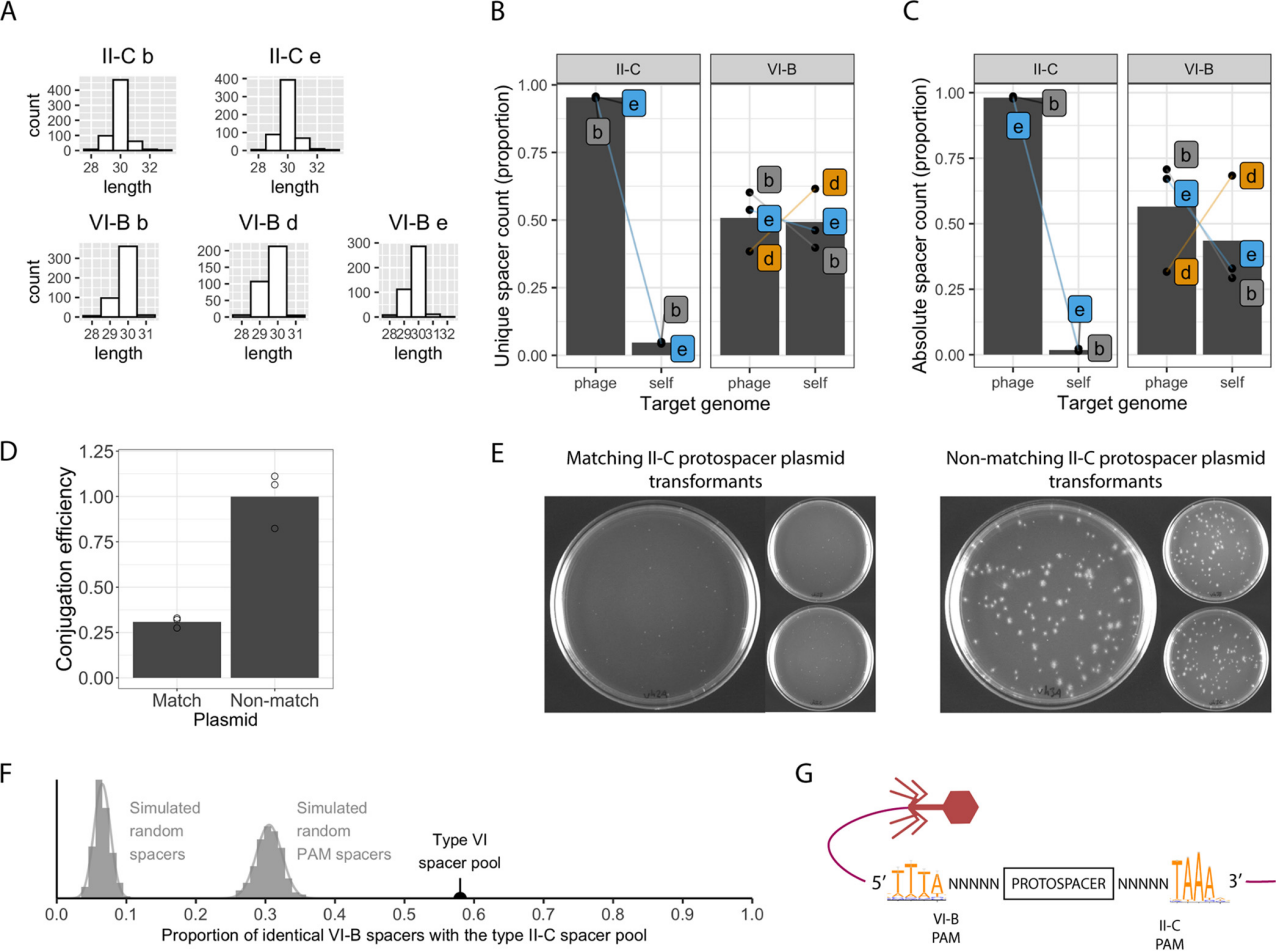
Analysis of acquired spacers in the coculture experiment. (A) Size distribution of unique spacers in different replicates. (B) Proportions of unique subtype II-C or VI-B spacers targeting the phage or bacterial (self) genome. Dots show the exact counts of spacers in a replicate (b, d, or e), and bars show their respective means. (C) Similar to panel B but showing proportions using absolute spacer counts. (D) Efficiency of conjugation (number of conjugants per recipient cell) normalized to the mean of the nonmatching control treatment. (E) *F. columnare* conjugants on antibiotic agar plates. Cells were conjugated with either a plasmid containing a protospacer matching the most recent II-C spacer or a plasmid containing a nonmatching control sequence. All three replicate plates per treatment are shown, with one replicate enlarged. (F) Proportions of simulated and observed VI-B spacers that are identical to the II-C spacer pool. (G) PAM sequences next to II-C and VI-B phage targets (sequences from the nontarget strand; example from replicate e) (for all replicates, see Fig. S2A in the supplemental material).

The lengths of new spacers varied between 28 and 32 bp, with the majority at 30 bp in both loci (Fig. 3A). We divided the new spacers into unique and absolute sets. In the unique spacer data set, each spacer sequence was counted only once, whereas the absolute set allowed for repeated observations of the same spacer. Most unique II-C spacers targeted the phage genome, with a minority targeting the bacterial genome (Fig. 3B). New VI-B spacers targeted both genomes relatively evenly, although the replicates had some differences (Fig. 3B). All differences were accentuated when the spacer count was viewed as absolute (Fig. 3C). The distinct targeting preferences between the loci are likely explained by the negative selection of autoimmunity resulting from Cas9-mediated self-targeting (e.g., see reference 53). The lowered fitness due to self-targeting in II-C is further implied by the proportional reduction of self-targeting spacers in the absolute data set (Fig. 3C) compared to the unique set (Fig. 3B). The abundance of self-targeting VI-B spacers was surprising given that such spacers could induce dormancy and be negatively selected (49). The observed pattern therefore indicates that, at least during this 7-day experiment, self-targeting VI-B spacers were not necessarily harmful.

10.1128/mBio.03338-20.2 FIG S2 PAM sequences from the unique spacer data set. (A) The 15-bp regions upstream and downstream of each protospacer displayed as WebLogos from replicates b and e for type II-C and replicates b, d, and e for type VI-B. PAMs were determined using the guide-oriented approach. (B) Variants of SAMs in both loci. Stacked bars show the proportions of different variants of SAMs in both loci and target genomes. Download FIG S2, TIF file, 1.0 MB. Copyright © 2021 Hoikkala et al. 2021 Hoikkala et al. https://creativecommons.org/licenses/by/4.0/ This content is distributed under the terms of the Creative Commons Attribution 4.0 International license.

To confirm that II-C spacers yield functional interference, we performed a plasmid interference assay. We modified plasmid pAS43 (54) to include a protospacer that matches the leader-adjacent II-C spacer in the conjugation-capable *F. columnare* strain B245. As a control plasmid, we replaced the matching protospacer sequence with a nonmatching sequence. Using Escherichia coli as the donor, the efficiency of conjugation was roughly three times lower in the matching protospacer treatment than in the control treatment under antibiotic selection (*P* = 0.0016; *t*_4_ = 7.62 [by an unpaired *t* test]) (Fig. 3D). Surviving colonies with the matching protospacer plasmid also had a reduced colony size (Fig. 3E). The II-C system in *F. columnare* seems to grant partial immunity to mobile genetic elements, leading to slower bacterial growth, at least when the CRISPR target is associated with positive selection (here, an antibiotic resistance-conferring plasmid). As speculated to occur with Listeria monocytogenes, small colonies may result from low Cas9 expression, allowing the persistence of a small number of plasmids in the cell (55).

To calculate the proportions of identical new spacers between the loci, we pooled unique phage-targeting spacers from both loci from all replicates. Remarkably, 58% of VI-B spacers were also found in the larger II-C spacer pool, suggesting that the loci share a predisposition toward a set of spacers. For statistical comparison, we simulated spacer acquisition across the phage genome by sampling random positions either freely or next to predicted PAM sites (5′-NNNNNTAAA-3′) (*n* = 1,528) (described in detail below). Both simulations were run 1,000 times, with each run sampling 430 spacer positions (the size of the VI-B spacer pool). We calculated the proportions of shared spacers between each run and the II-C spacers and found that on average, 6.3% (standard deviation [SD], 1.2%) of the random spacers and 29.6% (SD, 1.85%) of the PAM-adjacent spacers had a match in the subtype II-C spacer set (Fig. 3F). The similarity between the observed VI-B and II-C sets of spacers is significantly higher than that between either simulated pool and II-C spacers (*P* < 10^−10^ by a one-tailed test), which suggests that new spacers are sampled from a limited protospacer pool that is common to both loci.

### Shared acquisition motifs suggest in *trans* adaptation.

To further characterize the protospacers, we investigated PAM sequences for both loci. We used the guide-oriented approach that considers PAMs to be on the strand that matches a particular spacer’s crRNA (56), i.e., on the nontarget strand. It is necessary to establish the direction of crRNA transcription before evaluating the PAM sequence as the transcription direction determines the resulting crRNA strandedness. Previous studies have shown that unlike other CRISPR-Cas systems, subtype II-C arrays are expressed from within the array toward the variable end using repeat-encoded promoter sequences (5′-TAAAT-3′) (44, 48). The *F. columnare* II-C repeats lack this sequence but have a 5′-TTG-3′ motif 33 bp upstream of each spacer. As the sequence TTG is an established −33 promoter in Flavobacterium hibernum (57), this putative promoter may also drive II-C array transcription in *F. columnare.* This direction of transcription is also supported by our transcriptome data showing within-array transcripts that proceed toward the leader (albeit with only eight reads mapping on the array [Fig. S3A]). The subtype VI-B array, on the other hand, shows an abundance of transcripts occurring in the leader-to-array direction (Fig. S3B), as typically observed for CRISPR-Cas systems (11, 39), and has no putative repeat-encoded promoters.

10.1128/mBio.03338-20.3 FIG S3 Transcription directions of subtype II-C and VI-B arrays. The RNA samples were taken in the absence of phages to provide enough RNA for sequencing, leading to minimal yet sufficient expression of the arrays to determine the transcription direction. (a) Eight reads revealed type II-C transcription from within the array toward the leader (167.8 reads per million). (b) Forty-seven reads mapped on the type VI-B array revealed the transcription direction starting from the leader end (986.6 reads per million). The VI-B locus was reverse complemented from its genomic orientation for easier comparison. Download FIG S3, TIF file, 0.2 MB. Copyright © 2021 Hoikkala et al. 2021 Hoikkala et al. https://creativecommons.org/licenses/by/4.0/ This content is distributed under the terms of the Creative Commons Attribution 4.0 International license.

In subtype II-C, the previously reported PAM (34, 58) NNNNNTAAA was observed downstream of most (∼63%) phage-targeting spacers (Fig. 3G and Fig. S2A). In the few self-targeting subtype II-C spacers, the canonical PAM either was almost always absent or had extended or shortened N regions (Fig. S2B). This deprecation of the PAMs further suggests negative selection against functional self-targeting II-C spacers and indicates that both the nucleotide motif 5′-TAAA-3′ and the length of the N region in the PAM play a role in subtype II-C interference in *F. columnare* (Fig. S2B). The protospacers targeted by subtype VI-B had the same PAM sequence in the reverse complement (TTTANNNNN) and located upstream (Fig. 3G), with slightly less conservation (∼50%) than the subtype II-C protospacers (Fig. S2B). Self-targeting VI-B spacers did not have deprecated PAMs similar to the self-targeting II-C spacers, suggesting that PAMs do not affect VI-B functionality.

When using the guide-oriented approach, the PAMs of II-C and VI-B targets are complementary to each other and on opposite sides of the protospacer. For both loci, the PAM-adjacent ends of the spacers are oriented toward the leader. Therefore, while the target interference motif (TIM) of the loci is seemingly different, the spacer acquisition motif (SAM) is shared by the loci. The term TIM is not strictly applicable to the studied RNA-targeting type VI systems, and their interference efficiency has instead been shown to be affected by short protospacer-flanking sites (PFSs) (11, 39). Here, conserved PFSs were not discovered in the VI-B targets. Overall, these results suggest that the arrays likely share spacer acquisition machinery, as SAMs are generally hallmarks of specific acquisition complexes (12, 14).

### Spacer target distributions are nonuniform and show possible II-C priming.

**FIG 4:**
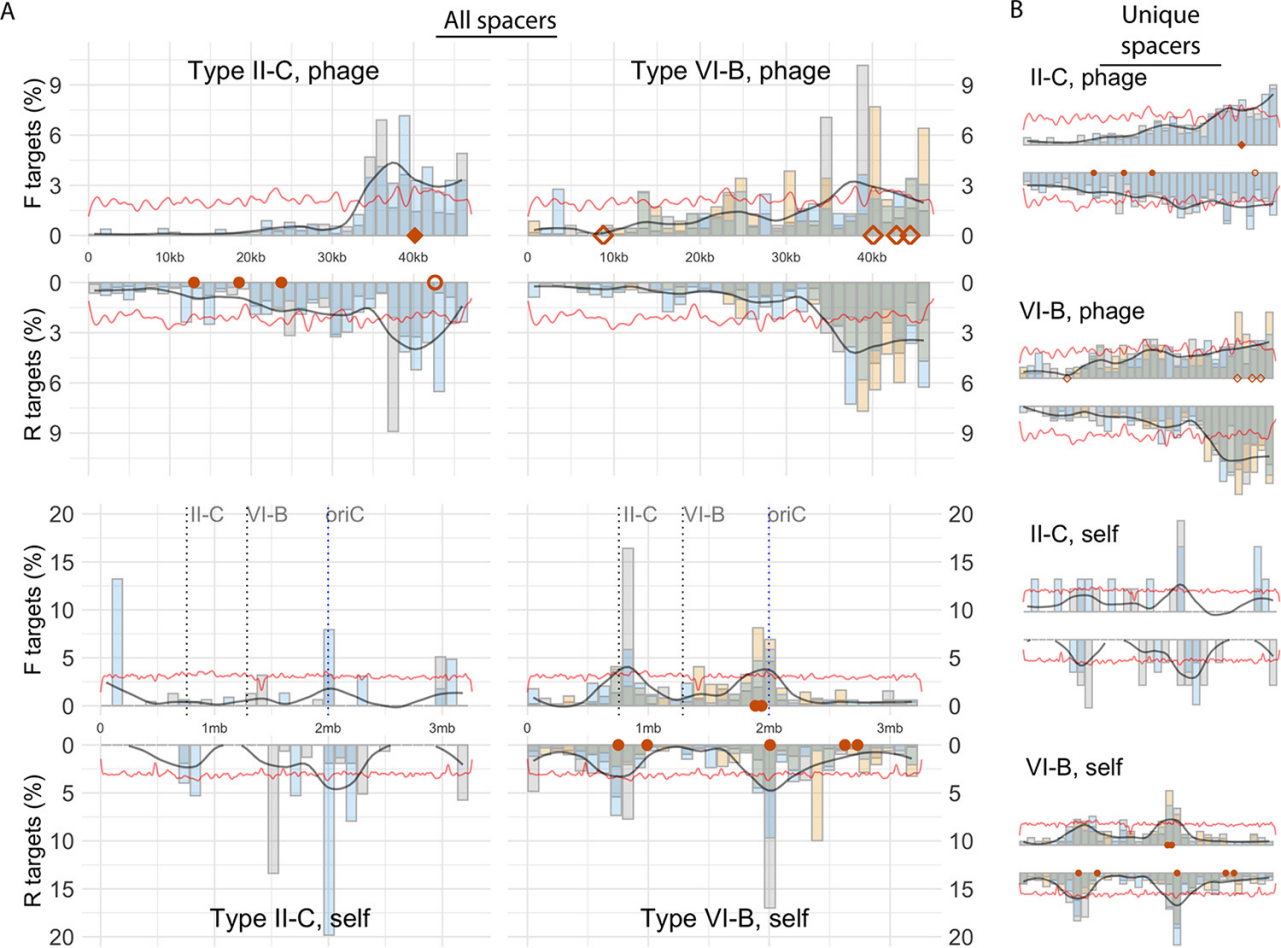
Distribution of spacers on the phage and bacterial genomes. The genomes are divided into bins (each spanning 3% of the genomes), whose proportions of spacer targets are shown on the *y* axes by bars (bar colors indicate different replicates, and “F targets” and “R targets” refer to the two DNA strands of the genomes). The black line indicates the smoothed average of targeting across replicates. The red line shows the relative frequency of the putative PAM sequence. Red markers are spacers that preexist in strain B185: diamonds target mRNA, and circles do not. Filled markers are spacers with perfect matches and PAMs, while nonfilled markers are spacers with 1- to 3-bp mismatches with their target sequence. (A) All spacers (absolute count) mapped on both genomes. (B) Unique spacers mapped on both genomes.

We investigated which regions of the genomes are targeted by II-C and VI-B spacers. To do this, we examined both unique and absolute spacer counts separately. Despite the relatively even distribution of predicted PAM sequences (5′-NNNNNTAAA-3′) across the phage genome (Fig. 4), spacer targets were not uniformly spread. On the linear phage genome, spacer targets were concentrated in the opposite end of the genome relative to the morphogenesis genes (59) and are therefore likely targeting the end of the genome that first enters the cell (Fig. 4). Furthermore, the two strands of the dsDNA phage genome were targeted unevenly, with stronger differences at the end of the genome (downstream of ∼30 kbp), especially in the absolute spacer set. These distributions are not explained by uneven PAM distributions on the phage genome (Fig. 4) or between the strands (the proportions of the 1,672 instances of the PAM sequence 5′-TAAA-3′ on the coding and template strands are 50.7% and 49.3%, respectively).

Intriguingly, the nonunique II-C spacers peak around a preexisting, fully matching II-C spacer on the phage genome (Fig. 4A) (around 40 kb), a trend not visible in the unique spacer set (Fig. 4B). The congregation of new spacers around a preexisting one may result from CRISPR-Cas type II primed adaptation (24), where spacers are acquired from the free dsDNA ends produced by Cas9 cleavage (25). Furthermore, the distribution of new spacers around this protospacer resembles an asymmetric pattern that is characteristic of primed adaptation in subtype I-F CRISPR-Cas systems (21, 22). It is unclear why the three additional phage-targeting II-C spacers (Table S3A) with canonical PAM sequences do not give rise to similar priming-like patterns (Fig. 4A). It is also unclear why the VI-B spacers do not form similar patterns, as the production of free dsDNA ends by Cas9 cleavage is expected to provide spacer material with no intrinsic bias toward any specific array.

Another possibility, not mutually exclusive with priming, is that targeting the terminal end of the linear phage genome that first enters the cell is predominant, as shown with the Staphylococcus pyogenes subtype II-A system (25). In phage FCL-2, the localization of the morphogenesis genes at the opposing end of the linear genome (59) suggests that the preferred targeted end is indeed the first to enter the cell. Natural samples of *F. columnare* also show consistent targeting of this end in other (genetically very similar) phage genomes (34). It is possible that primed adaptation and higher exposure to spacer sampling at this end of the phage genome cooccur, perhaps explaining why the three additional II-C spacers do not attract priming. In addition, if priming played no role in *F. columnare* adaptation, we would expect spacers to cluster at the very end of the genome instead of the currently observed priming-like peak approximately 10 kb from the end (Fig. 4A).

On the bacterial genome, VI-B spacers and the few II-C spacers congregated on two asymmetric hot spots (Fig. 4). One was centered on the subtype II-C CRISPR-Cas locus, while the other localized on the predicted origin of replication (oriC). Acquisition peaks on oriC were previously reported in self-targeting E. coli spacers, resulting from the frequent occurrence of double-stranded breaks in this region and the subsequent actions of the RecBCD dsDNA repair complex (60). The resulting prespacer substrates are overrepresented around oriC because this region is the first to be replicated and becomes enriched in a replicating cell. Acquisition peaks on CRISPR arrays were also documented previously (22, 60) and were speculated to arise from frequent nicking of DNA during spacer acquisition, as nicking of the chromosome would also stall the replication fork, leading to increased spacer acquisition (60). Why spacers do not congregate on the *F. columnare* VI-B locus is unclear.

### Most spacers are not targeting mRNAs.

To investigate if the new spacers reflect the different target requirements of the loci (DNA versus RNA), we examined the ability of each spacer crRNA to bind mRNA by assessing their complementarity to predicted open reading frames (ORFs) from both target genomes. Spacers from all replicates within a locus were also pooled for an overall estimate. For statistical context, we calculated the probabilities of mRNA-targeting proportions in both loci with a binomial distribution that uses sample sizes matching those of the pooled spacers and assumed an equal probability of targeting each strand. Deviation from these expected distributions signals possible bias toward or away from mRNA targeting.

**FIG 5:**
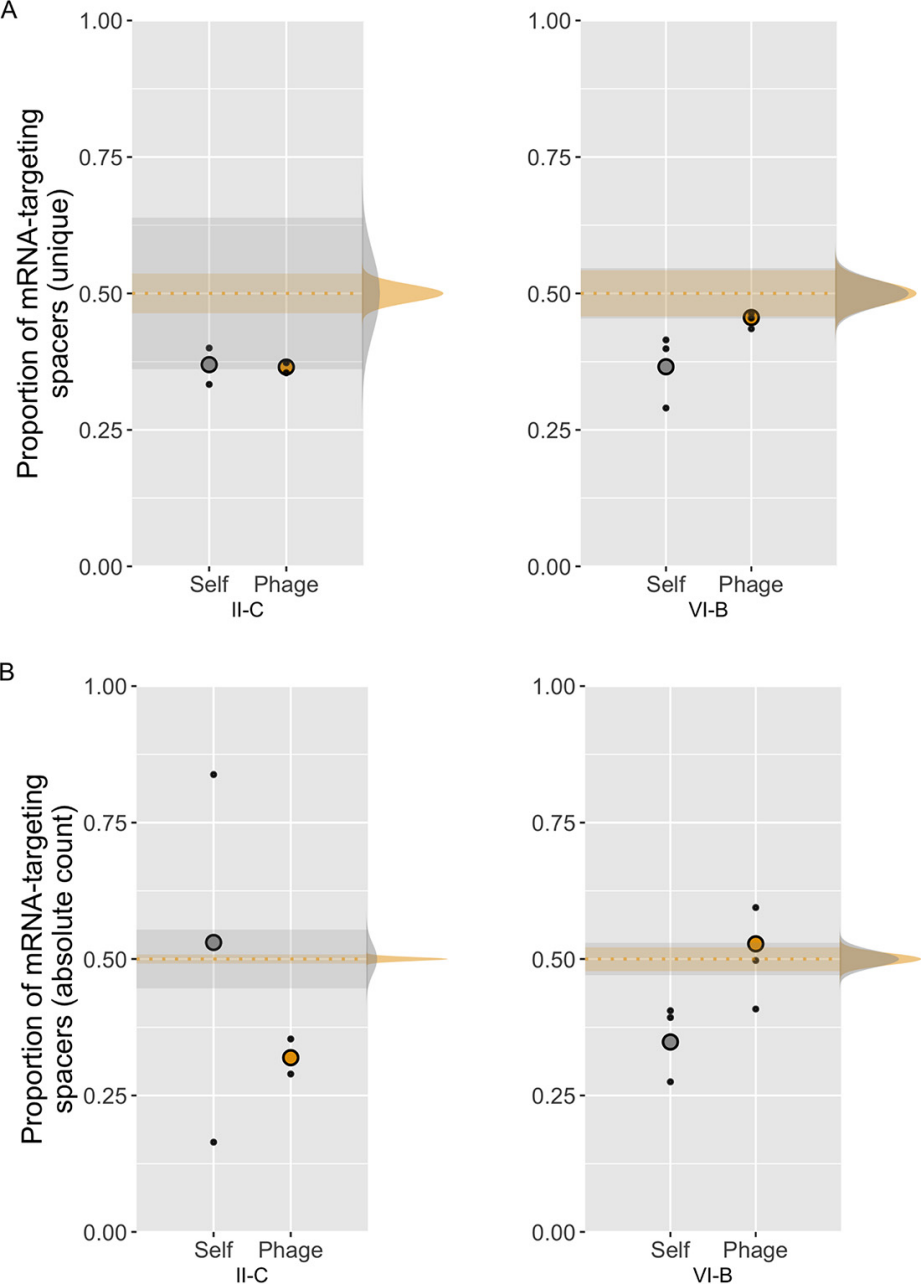
mRNA targeting in both CRISPR loci. *y* axes show the proportions of spacers complementary to predicted ORF mRNAs. Small dots are replicate cultures, and larger circles are the replicates pooled. Binomial distributions on the right side show the spread of expected values adjusted for the size of each spacer pool (yellow, phage; gray, host). The 95% confidence intervals of these expected values are projected on the graph with the same color coding. Deviations from these expected distributions suggest biased targeting. (A) Unique spacers. (B) Absolute spacer counts.

In the VI-B system, when considering unique spacers, the proportion of phage mRNA-targeting VI-B spacers was 0.46 (*P* = 0.074 by a one-tailed test), suggesting a weak or nonexistent bias toward non-mRNA-targeting spacers. VI-B mRNA targeting on the bacterial genome was rarer and significantly below expectation at 0.359 (*P* < 10^−9^ by a one-tailed test) (Fig. 5A). When considering the total spacer counts, the proportion of phage mRNA-targeting VI-B spacers rose to 0.53 (above expectation) (*P* = 0.0059 by a one-tailed test) (Fig. 5B), suggesting possible enrichment of mRNA-targeting spacers. On the bacterial genome, the mRNA-targeting proportions of absolute spacers were significantly below expectation at 0.348 (*P* = 10^−10^ by a one-tailed test) (Fig. 5B).

Subtype II-C mRNA-targeting proportions in the unique spacer set were the same for both target genomes (0.37 and 0.37). Given their vastly different sample sizes, the proportion of mRNA-targeting spacers on the phage genome was significantly below expectation (*P* < 10^−10^ by a one-tailed test), while self-targets were not clearly biased (*P* = 0.052 by a one-tailed test) (Fig. 5A). In the total spacer counts, the divergence of self-targeting II-C proportions may seem radical (Fig. 5B) but stem from the very small sample sizes, where a slight overrepresentation of a spacer may disproportionally skew the result by chance. Furthermore, the pooled II-C self-targeting spacers do not statistically deviate from expectation (*P* = 0.124 by a one-tailed test). The proportions of absolute phage-targeting II-C spacers were slightly lower than in unique counts, at 0.319, and deviated strongly from expectation (*P* < 10^−10^) (Fig. 5B).

The minimal phage mRNA targeting in subtype II-C spacers was unexpected, as Cas9 functionality is indifferent to which strand is being targeted. One potential explanation would be a strongly uneven distribution of PAM sequences on the coding versus template strands, but this is not the case in either genome: the proportions of PAM sequences (5′-TAAA-3′) in annotated coding sequences from B185 are 51.2%/48.8% (coding/template) (*n* = 81,490), and the proportions are 51.6%/48.4% for FCL-2 (*n* = 1,528). Another possibility is that competition between RNA and DNA targets for Cas9 association would impact subtype II-C immunity negatively.

The lack of new VI-B self-mRNA-targeting spacers is expected due to their possible dormancy-inducing effects (49). This is also in agreement with the lack of preexisting self-mRNA-targeting VI-B spacers in *F. columnare* B185 (*n* = 7) (Fig. 4 and Table S3B). However, this counterselection is much weaker than that against self-targeting II-C spacers (Fig. 3B and C), which argues for a lesser effect of VI-B autoimmunity in this experimental setting. Saturation of phage mRNA-targeting VI-B spacers was not observed here, which is likely due to the short time span of the experiment but may also be affected by the preexisting, fully matching II-C spacers (Table S3A). If the preexisting spacers already provide some level of CRISPR-Cas-based immunity, as suggested by our plasmid interference assay, the need to acquire new ones is decreased, and their selective advantage may be weaker.

Overall, the reasons for the unexpectedly low levels of mRNA-targeting spacers in both CRISPR loci remain speculative. In this analysis, we relied on predicted ORFs and do not take into account possible antisense transcription or intergenic transcripts; the number of RNA-targeting spacers may therefore be underestimated. In contrast, in our previous study on natural *F. columnare* isolates, there was a saturation of phage mRNA-targeting VI-B spacers (*n* = 19; *P* = 1.9 × 10^−6^ by a one-tailed binomial test) (including the preexisting four spacers in the wild-type array of B185 [Table S3B]). Our new results suggest that this was likely the result of positive selection toward mRNA-targeting spacers. Interference by the VI-B system in nature is also supported by the negative selection of targets: none of the preexisting VI-B spacers in strain B185 are complete matches with their protospacers on the phage genome, and VI-B protospacers accumulate mutations over time (34). Future studies are needed to assess how RNA-based VI-B immunity evolves and what constitutes functional targets for VI-B interference in native systems.

### The VI-B array uses II-C Cas1 in *trans.*

Identical spacer acquisition efficiencies, similar protospacer localization patterns, and shared PAM sequences supported the hypothesis that the *F. columnare* subtype II-C and VI-B CRISPR-Cas loci share spacer acquisition machinery. To verify this, we deleted *cas1* from the subtype II-C locus from *F. columnare* strain B245. Using the resulting B245 Δ*cas1* strain, we performed a prolonged adaptation experiment with 10 phage-exposed (phage V156) and 2 bacterium-only replicates. As a control, we used a “reversion wild-type” (rev-wt) strain that resulted from an alternative outcome of the mutation process and contains an intact *cas1* (see Materials and Methods). We did not observe spacer acquisition in either CRISPR array in any of the 12 Δ*cas1* cultures (Fig. S4), all of which survived until the end of the experiment. Of all 12 rev-wt cultures, only 2 phage-exposed replicates survived, both showing spacer acquisition in their II-C or VI-B arrays (Fig. S4). These results demonstrate the dependence of the VI-B locus on the II-C acquisition machinery.

10.1128/mBio.03338-20.4 FIG S4 Spacer acquisition experiment using the Δ*cas1* strain. (A) Subtype II-C array. (B) Subtype VI-B array. Ten replicates of Δ*cas1* and rev-wt cultures (annotated with numbers) were grown with phages along with two replicates without phages. Of the 12 rev-wt cultures, only 2 (replicates 1 and 5) had grown at the end of the 3-week experiment, both having acquired new spacers in the II-C and VI-B CRISPR-Cas loci. All Δ*cas1* replicates had grown by the end of the experiment, but none had acquired new spacers in either locus. The ladder used on gels is a 1-kb Plus DNA ladder (Thermo Fisher Scientific). Download FIG S4, TIF file, 0.7 MB. Copyright © 2021 Hoikkala et al. 2021 Hoikkala et al. https://creativecommons.org/licenses/by/4.0/ This content is distributed under the terms of the Creative Commons Attribution 4.0 International license.

The inability of B245 rev-wt to survive in the presence of phages is in contrast with the previous experiment performed with strain B185 and phage FCL-2. This difference may partly stem from the experimental setups, as the bacterial strains B245 and B185 were cultivated in different media and for 3 weeks and 1 week, respectively (see Materials and Methods). Also, strain B245 has four preexisting, fully matching phage mRNA-targeting spacers in the variable end of the VI-B arrays (Table S3C and D), whereas B185 has only incomplete VI-B spacer matches with its target, all located in the array’s conserved end. It is therefore possible that the B245 phage-targeting VI-B spacers induce dormancy during the CRISPR-Cas response (49), whereas B185’s lack of fully matching preexisting spacers does not. If this is the case, the survival of B245 Δ*cas1* is surprising as the inability to acquire new spacers should not abrogate interference using preexisting ones. These strains also belong to different genetic groups, and thus, their differences are not limited to spacer content. Further experiments to explore the effect of spacer diversity and the role of different *cas* genes and other host defenses in *F. columnare* are required.

### Comparison of II-C/VI-B leaders and repeats across species.

In *F. columnare*, subtypes VI-B and II-C have similarly sized repeats (36 bp), with sequence similarity mostly in the leader-distal ends (5′-GTTGT-3′) (Fig. 1). The lack of similarity in the leader-adjacent end of the repeat is surprising given the importance of this region during spacer insertion, at least in the S. thermophilus subtype II-A system (26). The *F. columnare* leaders also lack similarity in the leader-repeat junctions but share similarly positioned poly(A) and poly(T) regions up to 100 bp from the junction (Fig. 1). To see if II-C and VI-B CRISPR-Cas loci display similar patterns in other species, we extracted the repeats and leaders of nine species that carry these loci (lacking *cas1* and *cas2* in their VI-B locus). II-C and VI-B repeats shared the same leader-distal motifs across the species, but *F. columnare* was the only species with similarly sized II-C and VI-B repeats (II-C repeats in the other species were on average 11 bp longer) (Fig. S5). Leaders, clustered by Cas1 similarity for meaningful alignment, had leader-distal poly(A) and poly(T) motifs similar to those of *F. columnare* (Fig. S6), which may act as binding or signaling sites for spacer integration. As the generation of new repeats is based on a precise ruler mechanism in the acquisition complex (15, 27, 61), it remains unknown how the other species with differing repeat lengths generated their subtype VI-B arrays. It is therefore possible that all or some of the subtype VI-B CRISPR-Cas systems in these species are inactive and that *F. columnare* is one of the few or the only species engaging in cross talk between VI-B and II-C systems. Indeed, subtype II-C adaptation was recently shown in one of these species, Riemerella anatipestifer, but its cooccurring VI-B locus (denoted an orphan array) did not acquire new spacers (62).

10.1128/mBio.03338-20.5 FIG S5 Alignments of II-C and VI-B repeats from species that carry both loci. The 3′ ends are leader adjacent. Download FIG S5, TIF file, 0.2 MB. Copyright © 2021 Hoikkala et al. 2021 Hoikkala et al. https://creativecommons.org/licenses/by/4.0/ This content is distributed under the terms of the Creative Commons Attribution 4.0 International license.

10.1128/mBio.03338-20.6 FIG S6 Alignments of the II-C and VI-B leaders from species that carry both loci. The 5′ end is repeat adjacent, and open reading frames downstream of the leader are annotated with yellow rectangles. (A) Alignment of type II-C leaders (200 bp). (B) Alignment of type VI-B leaders (200 bp). (C to H) Alignments of both leaders within clusters. (I) Phylogenetic tree of Cas1 and the derived clusters. (J) Nucleotide similarity of leaders in the clusters. Download FIG S6, PDF file, 0.9 MB. Copyright © 2021 Hoikkala et al. 2021 Hoikkala et al. https://creativecommons.org/licenses/by/4.0/ This content is distributed under the terms of the Creative Commons Attribution 4.0 International license.

### *F. columnare* CRISPR-Cas model.

**FIG 6:**
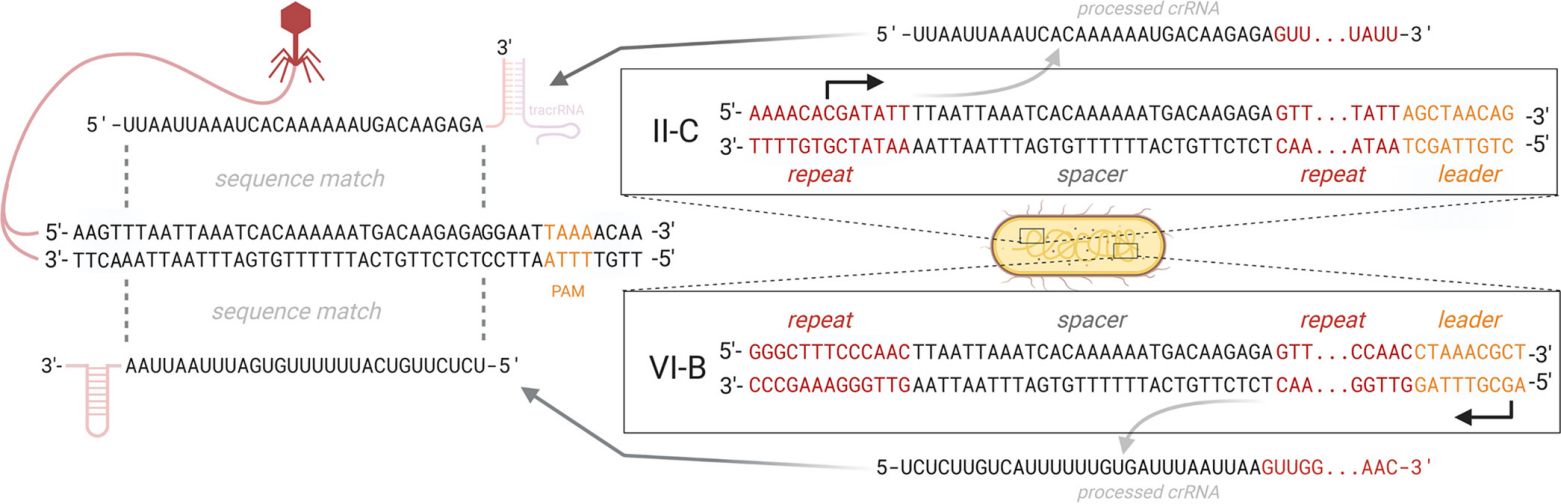
Model for subtype II-C and VI-B interference in *F. columnare*. In this scenario, the leader-adjacent spacer of the II-C array is also present in the VI-B array. However, due to opposite transcription directions, the crRNA is reversed and complementary between the loci. Mapping the crRNA onto the phage genome using the guide-oriented approach (56) reveals a downstream TIM for II-C and an upstream “TIM” for VI-B (orange letters on the phage genome), although the SAM is the same for both. All sequences shown here correspond to actual genomic sequences (except the VI-B spacer, which is the same as the II-C spacer for illustration purposes). The relative orders of spacers/repeats in crRNA are derived from previous studies (39, 48).

Based on our observations and previous biochemical studies, we propose a model for *F. columnare* spacer acquisition and interference. During acquisition, new spacers are captured by the Cas1/2 acquisition complex (possibly aided by Cas9) in a specific conformation relative to the PAM (63, 64). This prespacer is inserted into the array (15, 64–66), with the PAM end of the (proto)spacer facing the leader (6, 67, 68). Leader recognition is probably mediated by shared motifs in the subtype II-C and VI-B leaders and repeats that are distal from the leader-repeat junctions. During interference, spacers produce a downstream interference-PAM (TIM) in subtype II-C, as is generally observed in other type II systems (69). Since the arrays are transcribed in opposite directions with respect to the variable end of the array (Fig. 1 and Fig. S3), the TIM location is switched for subtype VI-B spacers during interference. Acquisition events relying on an identical SAM can therefore lead to two complementary interference patterns, dictated by the array in which the spacer is inserted (Fig. 6).

## DISCUSSION

We demonstrate that a native subtype VI-B CRISPR-Cas locus acquires spacers from a virulent dsDNA phage and from the host genome. Despite differences in repeat and leader sequences, shared characteristics of the newly acquired spacers with the coadapting subtype II-C locus support the model of in *trans* spacer acquisition in the subtype VI-B locus, which was confirmed by analysis of a *cas1* deletion mutant. This arrangement provides the RNA-targeting VI-B array with an abundance of spacers that do not target mRNA. Other species with similar CRISPR-Cas loci have more divergent repeat sequences, suggesting that in *trans* acquisition may not occur in these species or may utilize a highly plastic acquisition complex. This study also highlights *F. columnare* as one of the few known species that have been demonstrated to acquire spacers from virulent phages under laboratory conditions (70).

Our conjugation assay suggested functional yet limited interference efficiency of the II-C locus against plasmids, with a 3-fold reduction in conjugation efficiency. Similarly, our previous study (34) showed that preexisting spacers do not grant *F. columnare* efficient phage immunity. These results may be explained by suboptimal experimental conditions (i.e., high nutrient/phage concentrations) for testing interference. Another possibility for low efficiency against phages may be phage-encoded anti-CRISPR proteins (71). On the other hand, the lack of self-targeting II-C spacers in this study suggests effective autoimmunity.

While our plasmid assay did not address VI-B interference, its role in actively targeting mRNA can be deduced by contrasting naturally acquired VI-B spacers with those acquired in the laboratory: both mRNA- and DNA-targeting VI-B spacers were acquired in the current study, but only mRNA-targeting spacers are observed in natural samples of *F. columnare* (34), suggesting positive selection of such spacers under natural conditions. Also, none of the preexisting VI-B spacers of *F. columnare* B185 (see Table S3B in the supplemental material) are perfect matches with their targets, suggesting that phages have previously escaped VI-B targeting, as also suggested by multiple natural isolates (34).

We expect future studies to reveal the extent of cross talk in type VI systems and the biochemical basis for the plasticity of the acquisition machinery when interacting with different leader and repeat sequences. Possible priming in the II-C locus should also be studied in detail, as should the interference stage of both loci, including the possible dormancy-generating effects of the VI-B locus.

## MATERIALS AND METHODS

### Bacterial cultures and sampling.

Two strains were used to test for CRISPR spacer acquisition: Flavobacterium columnare B185 and B245 (34). Strain B185 was used as the host in the main spacer acquisition assays, and strain B245 was used in the *cas1* deletion mutant experiments. Strain B185 was revived from a freezer stock in a Shieh medium culture grown overnight (72). Five replicates of 5-ml cultures were then inoculated in 0.1× Shieh medium with the culture grown overnight to produce an initial concentration of 10^4^ CFU/ml. Phage FCL-2 (34) was added to the samples at a multiplicity of infection (MOI) of 1. For the phage UV treatment (52), phage FCL-2 was exposed to UV light for 5 min on a petri dish (5,000 μJ) (UV Stratalinker 1800; Stratagene), lowering the infectivity by roughly 2 orders of magnitude (data not shown). In the bacterium plus phage plus UV-phage treatments, wild-type phages were mixed with an equal volume of UV-treated phages from the same stock and dilution. Five bacterium-only and bacterium plus UV-phage cultures were established as controls. The cultures were incubated under agitation (120 rpm) at room temperature for 5 days, after which they were transferred (1:100) to 5 ml of 1× Shieh medium. After 2 days (day 7 from the beginning of the experiment), cell debris had sedimented at the bottom of the tubes. We sampled 1 ml of the clear phase for living planktonic cells and extracted DNA using a DNeasy blood and tissue kit (Qiagen). The cultures were also plated on Shieh agar plates with 10-fold dilutions to estimate CFU per milliliter and to perform colony PCR.

### Colony PCR.

The variable ends of subtype II-C and VI-B CRISPR arrays were PCR amplified from colonies obtained under all conditions (total of 281 colonies) at day 7 (see Table S1A in the supplemental material) using primers C1_B185_F and C1_B185_R and primers C2_B185_F and C2_B185_R (Table S2A). Colonies with expanded arrays were recultured on Shieh agar plates, and the variable ends of the CRISPR arrays were rechecked. Colonies that still showed expanded arrays were grown in liquid medium, the DNA was extracted with a blood and tissue kit (Qiagen), and the variable ends were sequenced with the Sanger method using a BigDye Terminator v3.1 cycle sequencing kit (Applied Biosystems) and the automated 3130xl genetic analyzer (Applied Biosystems) (Table S1B).

### Population-level CRISPR deep sequencing.

Total DNA was extracted from 1-ml liquid samples using a DNeasy blood and tissue kit (Qiagen). The variable ends of subtype II-C and VI-B CRISPR loci (C1 and C2, respectively) from the phage and bacterium-only treatments were amplified with DreamTaq (Thermo Fisher Scientific), with one primer binding to the leader sequence (C2_B185_F and C1_B185_R) and one binding to the second (C1_B185_F) or third (C2_B185_R) spacer (Table S2A). The PCR protocol to amplify the CRISPR array associated with subtype II-C was as follows: 95°C for 3 min; 32 cycles of 95°C for 30 s, 59°C for 30 s, and 72°C for 1 min; and 72°C for 15 min. The PCR protocol to amplify the CRISPR array of subtype VI-B was as follows: 95°C for 3 min; 30 cycles of 95°C for 30 s, 60.2°C for 30 s, and 72°C for 1 min; and 72°C for 15 min. For subtype II-C, additional MgCl_2_ (4 mM) was required for amplification. To minimize PCR bias, four separate PCRs were performed for each of the five replicates, which were then pooled and cleaned using a Qiagen MinElute reaction cleanup kit. The resulting 10-μl samples were run on a 2% agarose gel (4.6 V/cm) for 2 h 45 min in Tris-acetate-EDTA (TAE) buffer. The expected PCR product sizes for the wild-type array (subtype II-C, 181 bp; subtype VI-B, 223 bp) as well as for the expanded arrays with one new repeat-spacer unit (subtype II-C, 246 bp; subtype VI-B, 289 bp) were extracted from the phage treatment using X-tracta (Sigma) extraction tools and a gel extraction kit (Qiagen) using MinElute columns (Qiagen). In the bacterium-only treatments, the wild-type PCR products from both loci were extracted as controls. All extractions underwent two gel purification rounds to reduce contamination.

For deep sequencing, we used the previously published pipeline for multiplexed Ion Torrent sequencing (4). The extracted PCR products from type VI-B were reamplified using Maxima Hot Start *Taq* DNA polymerase (Thermo Fisher Scientific), the primers M13-B185_223bp_C2F and P1-B185_223bp_C2R, as well as the IonA_bc_M13 primer that contained multiplexing barcodes and an M13 adaptor (Table S2A) (73). The following PCR program was used: 95°C for 5 min; 20 cycles of 94°C for 45 s, 53°C for 1 min, and 72°C for 1 min; and 72°C for 5 min. As we were unable to obtain amplification of the type II-C array with the above-described three-primer PCR approach, the PCR was split into two stages. First, type II-C PCR products were amplified using the primer pair M13-B185_C1_F3/P1-B185_181bp_C1R and the DreamTaq enzyme with added MgCl_2_ (4 mM) using the following program: 95°C for 5 min; 31 cycles of 95°C for 30 s, 59°C for 30 s, and 72°C for 1 min; and 72°C for 15 min. The reaction mixture was then purified with Agencourt AMPureXP (Beckman Coulter) and reamplified using primers IonA-bc_M13 (73) and P1-B185_181bp_C1R in a reaction identical to the three-primer reaction used for type VI-B. Finally, all samples were purified using AMPureXP and quantified with a Qubit fluorometer and a Qubit dsDNA HS kit. Equimolar amounts (5 ng) of PCR products were pooled for sequencing. Pooled PCR products were sequenced after emulsion PCR with the Ion OneTouch system and Ion OT2 400 kit (Life Technologies) on Ion 314 chips with the Ion PGM (personal genome machine) sequencing 400 kit (Life Technologies), according to the manufacturer’s instructions.

### Deep sequencing data preparation.

Nontrimmed reads were obtained from PGM and sorted by their barcodes. Trimmomatic 0.36 (74) was used for quality control with the following parameters: SLIDINGWINDOW:3:21, MINLEN:100, and TRAILING:23. Spacers were extracted from the trimmed reads using a custom Python script that extracted 27- to 32-bp spacers between an intact first repeat and the first 4 nucleotides of the subsequent repeat. Due to short reads, a fully intact second repeat was usually not available. To obtain unique spacers, each spacer pool was clustered with CD-hit-est (75) using a clustering threshold of 0.8 and a word size of 5. The resulting spacers before and after filtering are listed in Table S2B.

### Mapping.

The spacers were mapped to the previously published phage genome (59) using Bowtie 2.0 (76) with the following custom configuration: –very-sensitive-local –score-min G,10,8. The physical ends of the linear phage genome had been determined previously using a combination of next-generation sequencing and Sanger sequencing (59). Unmapped spacers were then mapped onto the bacterial genome, and the remaining unmapped reads (most probably due to poor sequence quality) were discarded. On average, 7.9% and 1.14% of unique spacers were discarded in the type II-C and VI-B loci, respectively. The genomes were divided into bins that span 3% of the respective genomes, and the protospacer count of each bin was calculated with a custom Python script. The resulting protospacer distribution on both genomes was illustrated using the ggplot2 package in RStudio 1.1.463 (R 3.5.3). The predicted origin of replication (oriC) region of *F. columnare* was determined using DoriC 10.0.0 (77).

### PAM sequences.

With the aim of comparing PAM sequences between the loci, we report all PAM sequences based on flanking DNA (instead of RNA) regions due to the large proportion of only-DNA-targeting type VI-B spacers. Similarly, we follow the guide-oriented approach for both loci, as is common with DNA-targeting systems (56) (PAM is depicted on the noncomplementary strand with respect to the crRNA). All PAM sequences were extracted using custom Python scripts and shown using WebLogo (78).

### Spacer pool identity level analysis.

To calculate the proportion of subtype VI-B spacers that are identical to II-C spacers, unique VI-B spacers from all replicates (b, d, and e) were first pooled. CD-hit-est was used to extract unique spacers from the pooled spacer set (clustering threshold of 0.8). The process was repeated for the type II-C spacers from replicates b and e. Next, the pooled unique spacer sets from both loci were compared to each other with CD-hit-est-2D using a similarity threshold of 0.9. The number of resulting clusters was then used to calculate the proportion of VI-B spacers that had a match in the type II-C spacer pool. Simulated sets of 430 spacers (the number of unique VI-B spacers pooled from all three replicates) were generated by sampling the phage genome either at random positions or from the 1,672 predicted PAM sites (5′-NNNNNTAAA-3′). Sampling was repeated 1,000 times, and similarities of the simulated spacers were compared with the type II-C spacer pool using CD-hit-est-2D as described above. A normal distribution was fit onto the simulated distributions using the R function fitdistr from the MASS package. The probability of the observed similarity, given the null hypothesis, between subtype II-C and VI-B spacers (0.58) was measured using the function pnorm from the upper tails of the distributions.

### Proportions of mRNA-targeting spacers.

The ability of each spacer to target an ORF’s transcript was determined by two rules: (i) the crRNA of the spacer must be complementary to the coding strand, and (ii) the protospacer must be fully contained within an ORF. Intergenic spacers were excluded from the analysis. The number of mRNA-targeting spacers was divided by the total number of ORF-targeting spacers in the sample to obtain the proportion of mRNA-targeting spacers for each replicate separately. We also performed the analysis for pooled spacers from the replicates. Pools were made on the basis of locus and target, resulting in four pools (II-C phage, II-C self, VI-B phage, and VI-B self).

The mRNA proportions from pooled spacers were compared to a null hypothesis that assumed an equal chance of a spacer targeting both strands. Since intergenic spacers were excluded from the analysis, a binomial distribution could be used to construct the model. Separate distributions were created for each spacer pool to account for different numbers of spacers in each. The observed values in both loci were then compared to their respective distributions to yield direct *P* values on their probability given the null hypothesis described above (one-tailed test using the pbinom function in R). This analysis was done separately for the unique and absolute spacer counts.

### RNA-seq and transcription direction.

The direction of CRISPR array transcription was determined from bacterial RNA sequencing (RNA-seq) data. *F. columnare* B185 was grown without phages in 10 ml of Shieh medium at 24°C with constant shaking at 150 rpm. Next, 24-h-grown cultures (optical density [OD] of 0.166 to 0.203) were centrifuged (5,000 rpm) and stored in RNAlater (Qiagen), until RNA was extracted with an Ambion MicrobExpress mRNA purification kit. RNA quality was verified using an Agilent Bioanalyzer 2100 RNA nanochip, and samples with RNA integrity values of >9.5 were selected for library preparation (Ion Total RNA-seq kit v2). The cDNA was sequenced with Ion Torrent using a 318 chip (v2) and an internal ERCC (External RNA Controls Consortium) spike-in control, after ensuring cDNA quality (Agilent Bioanalyzer 2100 DNA high-sensitivity chip). Our results support previous reports showing type VI-B crRNA transcription from the leader end (39) and type II-C transcription toward the leader end (starting from within each repeat) or the leader-distal end of the array (48) (Fig. S3). The RNA samples for this analysis were taken from three pooled cultures of *F. columnare* B185 in the absence of phages, showing that these arrays are expressed constitutively albeit at a very low level (reads mapping on arrays at 167.8 and 986.6 reads per million in II-C and VI-B, respectively).

### *cas1* deletion mutant.

Not all *F. columnare* strains accept plasmids by conjugation (54). As strain B185 was unable to receive plasmids via conjugation or electroporation (data not shown), we used strain B245 (34) to create the *cas1* deletion mutant Δ*cas1*. Due to its competence in plasmid conjugation, strain B245 was also used in the plasmid interference assay described below. The 2.1-kbp region upstream of *cas1* was amplified by PCR using Phusion DNA polymerase (New England BioLabs) and primers 2322 (adding a KpnI site) and 2323 (adding a BamHI site). The PCR product was digested with KpnI and BamHI and ligated into the plasmid pMS75 (79) that had been digested with the same enzymes, to produce pRC30. A 495-bp region downstream of *cas1* was PCR amplified using primers 2324 (adding a BamHI site) and 2325 (adding a SalI site) (Table S2A). The PCR product was digested with BamHI and SalI and ligated into pRC30 that had been digested with the same enzymes, to generate pRC32. The plasmid pRC32 was transferred from E. coli S17-1λpir into *F. columnare* strain B245 by conjugation. One milliliter of a culture of the recombinant E. coli strain grown overnight was inoculated into 9 ml of LB containing 100 μg/ml ampicillin and incubated with shaking at 37°C until the OD at 600 nm (OD_600_) reached 0.6. Similarly, 5 ml of a culture of *F. columnare* B245 grown overnight was inoculated into 25 ml fresh TYES (tryptone yeast extract salts) broth (80) and incubated at 28°C with shaking, until the OD_600_ reached 0.6. The E. coli and *F. columnare* cells were centrifuged separately at 5,000 rpm for 15 min, and the pellets were washed with 10 ml of TYES medium and centrifuged at 5,000 rpm for 10 min. The E. coli and *F. columnare* cell pellets were each suspended in 0.8 ml of TYES medium, mixed, and centrifuged at 7,000 rpm for 3 min. Excess medium was removed, and the mixed pellet was suspended and spotted on FCGM (Flavobacterium columnare growth medium) agar medium (80). After incubation at 30°C for 24 h, cells were scraped off the plate and suspended in 1.5 ml of TYES medium. Next, 100-μl aliquots were spread on TYES agar containing 1 μg/ml tobramycin and 5 μg/ml tetracycline and incubated at 30°C for 72 h. The resulting tetracycline-resistant colonies were streaked for isolation, inoculated into TYES liquid medium without tetracycline, and incubated overnight at 25°C with shaking to allow plasmid loss. The cells were plated on TYES medium containing 10% sucrose, incubated at room temperature (20°C) to select for the lack of sucrose toxicity, and streaked for isolation using the same selection. The *cas1* deletion was screened by PCR and gel electrophoresis and verified with Sanger sequencing using primers Cas1_F and Cas1_R. All primers used are listed in Table S2A.

Strains used in the new spacer acquisition experiment were Δ*cas1* and B245_rev (rev-wt). The latter is a reversion mutant where the integrated plasmid was lost by recombination in a manner that regenerated the wild-type sequence. Phage V156 (34) was used to trigger spacer acquisition. The infectivity of phage V156 against the two hosts (Δ*cas1* and rev-wt) was measured with a standard double-layer method, where 300 μl of a turbid bacterial culture was mixed with 3 ml of TYES medium with 0.1% mucin and 1% agar. The mixture was poured onto a TYES agar plate, and 10 μl of phage V156 dilutions (10^−1^ to 10^−7^) were spotted in duplicates onto the solidified agar. The number of plaques was counted after 2 days from the 10^−7^ dilutions. Phage titers (PFU per milliliter) were 1.9 × 10^10^ and 3.7 × 10^10^ on the Δ*cas1* and rev-wt strains, respectively.

For the spacer acquisition experiment, both strains were grown overnight in single 5-ml cultures to reach an OD_600_ of 0.45. Ten phage-infected cultures in 0.1× TYES medium as well as two bacterium-only cultures were started from these cultures grown overnight. The spacer acquisition protocol differed from the one used with B185, as follows: medium TYES (versus Shieh medium), volume of 1 ml (versus 5 ml), and duration of growth in 0.1× medium of 3 weeks (versus 5 days). The protocol was prolonged because follow-up experiments showed that longer incubation in diluted medium resulted in more efficient spacer acquisition. The medium or its volume has no detectable effect on the acquisition efficiency (data not shown) and was changed for practical reasons. After 3 weeks in diluted medium (0.1×), the cultures were transferred to regular TYES medium (1×), and the CRISPR arrays were PCR amplified after 2 days. All 12 phage-containing cultures of the Δ*cas1* strain showed growth in 1× medium, in contrast to only 2 of 12 replicates of the rev-wt cultures. The variable ends of both CRISPR arrays in all surviving cultures were PCR amplified using primers B245_C1_F and F_col_C1_R (B245 II-C array) and primers F_col_C2_F and B245_C2_R (B245 VI-B array) (Table S2A and Fig. S4).

### Plasmid interference assay.

A derivative of plasmid pAS43 (54) (with a removed KpnI restriction site, not relevant to this experiment) was used as a template for adding a protospacer sequence followed by the putative PAM sequence 5′-NNNNNTAAA-3′. Two plasmids were constructed: one with a protospacer matching the most recent II-C spacer (next to the leader sequence) of *F. columnare* strain B245 (GGTAATTTTAAAACAAATGAGTATGTACGAACTGCTAAA [the PAM sequence is underlined]) and one with a nonmatching control sequence (ATCAGATCTAATCTCTATGTCAATGTATGAACTGCTAAA [the PAM sequence is underlined]). The insertions were added to the plasmid at an intergenic multiple-cloning-site region using the Q5 site-directed mutagenesis kit (New England BioLabs) and the oligonucleotide pair pAS43_protoSDM_F/R (matching protospacer) or pAS43_protoSDM_neg_F/R (nonmatching protospacer) (Table S2A). The initial PCR was done using Phusion polymerase with 3% dimethyl sulfoxide (DMSO) using the following program: 98°C for 10 s and 24 cycles of 98°C for 1 s, 60°C for 7 s, 72°C for 12 min, and 72°C for 15 min. The amplified product was inspected on an agarose gel and subjected to downstream KLD (kinase, ligase and DpnI mix) treatment according to the mutagenesis kit instructions. The resulting plasmid was transformed into chemically competent E. coli strain S17-1λpir. Successful transformation and insertion were verified by colony PCR and Sanger sequencing (primer pair pAS43_MCS2_seq_F/R) (Table S2A).

In the quantitative conjugation assay, the plasmids were transferred into *F. columnare* strain B245. Both the donor and the recipient cells were first grown overnight from a freezer stock. The turbid cultures were then transferred to fresh medium (LB for E. coli and TYES medium for *F. columnare*). E. coli was grown to an OD_600_ of 0.95, and the recipient *F. columnare* strain was grown to an OD_600_ of 0.414. The conjugation protocol was performed similarly to the *cas1* deletion (described above), and the final donor/recipient mixtures were spotted on 0.45-μm filters resting on FCGM plates. After 24 h of growth at 30°C, the cells were scraped from the filters and resuspended in 2 ml TYES liquid medium. Next, 150 μl of the mixed cultures was plated on double-antibiotic selection plates without dilution (cefoxitin for conjugant selection and tobramycin for eliminating E. coli) as well as on tobramycin-only plates with 10^−5^ and 10^−6^ dilutions for determining the number of potential recipient cells in each replicate (*F. columnare* is naturally resistant to tobramycin). Colonies were counted and plates were photographed after 72 h at 25°C. The efficiency of conjugation was determined by dividing the number of *F. columnare* conjugants (double-selection plate) by the number of potential recipients (tobramycin plates) in each replicate.

### Comparison of type II-C and VI-B leaders and repeats in other species.

Microbial species that carry intact subtype II-C (containing *cas1*, *cas2*, *cas9*, and a CRISPR array) and VI-B (containing *cas13b* and a CRISPR array) loci were identified using CRISPRCasdb (81), and one strain per species was selected for further analysis (Flavobacterium branchiophilum was excluded from the analysis due to the absence of *cas1* in the subtype II-C locus). From each strain, repeat sequences from both loci were aligned with Geneious aligner (global alignment with free gaps, 51% cost matrix, gap open penalty of 12, and gap extension penalty of 3). Next, 200-bp leader sequences were extracted downstream of the expected variable end of the array. The variable ends were primarily determined by the repeats’ direction compared to *F. columnare* strain B185. These initial predictions were supported by the presence of possible degenerate terminal repeats and alignment scores of both flanking regions compared to the leader of *F. columnare* B185. To allow the comparison of the highly divergent leaders, the strains were first clustered based on their Cas1 protein (Jukes-Cantor, unweighted pair group method using average linkages [UPGMA]). The leaders from both loci within the resulting six clusters (most of which contained single species) were aligned with Geneious aligner (global alignment, 65% cost matrix, gap open penalty of 17, and gap extension penalty 12). We used strict alignment rules to emphasize the importance of the distance of possible motifs from the repeat-leader junction. Analyses and alignments were performed with Geneious 9.1.8.

### Data availability.

The B245 genome and raw reads from the spacer acquisition experiment have been submitted to GenBank under accession numbers CP071008 and SAMN18022999, respectively. The genomes of *F. columnare* strain B185 (accession no. NZ_CP010992.1) and phage FCL-2 (accession no. NC_027125.1) are available in GenBank. Custom code and instructions for analyzing the raw data and reproducing all figures in the manuscript are in GitHub at https://github.com/vihoikka/spacerAQ_vh.

## References

[1] Mojica FJ, Díez-Villaseñor C, Soria E, Juez G. 2000. Biological significance of a family of regularly spaced repeats in the genomes of Archaea, Bacteria and mitochondria. Mol Microbiol 36:244–246. doi:10.1046/j.1365-2958.2000.01838.x. 10760181

[2] Jansen R, van Embden JDA, Gaastra W, Schouls LM. 2002. Identification of genes that are associated with DNA repeats in prokaryotes. Mol Microbiol 43:1565–1575. doi:10.1046/j.1365-2958.2002.02839.x. 11952905

[3] Barrangou R, Fremaux C, Deveau H, Richards M, Boyaval P, Moineau S, Romero DA, Horvath P. 2007. CRISPR provides acquired resistance against viruses in prokaryotes. Science 315:1709–1712. doi:10.1126/science.1138140. 17379808

[4] Makarova KS, Wolf YI, Iranzo J, Shmakov SA, Alkhnbashi OS, Brouns SJJ, Charpentier E, Cheng D, Haft DH, Horvath P, Moineau S, Mojica FJM, Scott D, Shah SA, Siksnys V, Terns MP, Venclovas Č, White MF, Yakunin AF, Yan W, Zhang F, Garrett RA, Backofen R, van der Oost J, Barrangou R, Koonin EV. 2020. Evolutionary classification of CRISPR-Cas systems: a burst of class 2 and derived variants. Nat Rev Microbiol 18:67–83. doi:10.1038/s41579-019-0299-x. 31857715 PMC8905525

[5] Horvath P, Romero DA, Coûté-Monvoisin A-C, Richards M, Deveau H, Moineau S, Boyaval P, Fremaux C, Barrangou R. 2008. Diversity, activity, and evolution of CRISPR loci in *Streptococcus thermophilus*. J Bacteriol 190:1401–1412. doi:10.1128/JB.01415-07. 18065539 PMC2238196

[6] Mojica FJM, Díez-Villaseñor C, García-Martínez J, Almendros C. 2009. Short motif sequences determine the targets of the prokaryotic CRISPR defence system. Microbiology (Reading) 155:733–740. doi:10.1099/mic.0.023960-0. 19246744

[7] Deveau H, Barrangou R, Garneau JE, Labonté J, Fremaux C, Boyaval P, Romero DA, Horvath P, Moineau S. 2008. Phage response to CRISPR-encoded resistance in *Streptococcus thermophilus*. J Bacteriol 190:1390–1400. doi:10.1128/JB.01412-07. 18065545 PMC2238228

[8] Hale CR, Zhao P, Olson S, Duff MO, Graveley BR, Wells L, Terns RM, Terns MP. 2009. RNA-guided RNA cleavage by a CRISPR RNA-Cas protein complex. Cell 139:945–956. doi:10.1016/j.cell.2009.07.040. 19945378 PMC2951265

[9] Jinek M, Chylinski K, Fonfara I, Hauer M, Doudna JA, Charpentier E. 2012. A programmable dual-RNA-guided DNA endonuclease in adaptive bacterial immunity. Science 337:816–821. doi:10.1126/science.1225829. 22745249 PMC6286148

[10] Gasiunas G, Barrangou R, Horvath P, Siksnys V. 2012. Cas9-crRNA ribonucleoprotein complex mediates specific DNA cleavage for adaptive immunity in bacteria. Proc Natl Acad Sci U S A 109:E2579–E2586. doi:10.1073/pnas.1208507109. 22949671 PMC3465414

[11] Abudayyeh OO, Gootenberg JS, Konermann S, Joung J, Slaymaker IM, Cox DBT, Shmakov S, Makarova KS, Semenova E, Minakhin L, Severinov K, Regev A, Lander ES, Koonin EV, Zhang F. 2016. C2c2 is a single-component programmable RNA-guided RNA-targeting CRISPR effector. Science 353:aaf5573. doi:10.1126/science.aaf5573. 27256883 PMC5127784

[12] Kieper SN, Almendros C, Behler J, McKenzie RE, Nobrega FL, Haagsma AC, Vink JNA, Hess WR, Brouns SJJ. 2018. Cas4 facilitates PAM-compatible spacer selection during CRISPR adaptation. Cell Rep 22:3377–3384. doi:10.1016/j.celrep.2018.02.103. 29590607 PMC5896167

[13] Shah SA, Erdmann S, Mojica FJM, Garrett RA. 2013. Protospacer recognition motifs. RNA Biol 10:891–899. doi:10.4161/rna.23764. 23403393 PMC3737346

[14] Heler R, Samai P, Modell JW, Weiner C, Goldberg GW, Bikard D, Marraffini LA. 2015. Cas9 specifies functional viral targets during CRISPR-Cas adaptation. Nature 519:199–202. doi:10.1038/nature14245. 25707807 PMC4385744

[15] Kim JG, Garrett S, Wei Y, Graveley BR, Terns MP. 2019. CRISPR DNA elements controlling site-specific spacer integration and proper repeat length by a type II CRISPR-Cas system. Nucleic Acids Res 47:8632–8648. doi:10.1093/nar/gkz677. 31392984 PMC6895254

[16] Silas S, Mohr G, Sidote DJ, Markham LM, Sanchez-Amat A, Bhaya D, Lambowitz AM, Fire AZ. 2016. Direct CRISPR spacer acquisition from RNA by a natural reverse transcriptase-Cas1 fusion protein. Science 351:aad4234. doi:10.1126/science.aad4234. 26917774 PMC4898656

[17] Toro N, Mestre MR, Martínez-Abarca F, González-Delgado A. 2019. Recruitment of reverse transcriptase-Cas1 fusion proteins by type VI-A CRISPR-Cas systems. Front Microbiol 10:2160. doi:10.3389/fmicb.2019.02160. 31572350 PMC6753606

[18] Yosef I, Goren MG, Qimron U. 2012. Proteins and DNA elements essential for the CRISPR adaptation process in *Escherichia coli*. Nucleic Acids Res 40:5569–5576. doi:10.1093/nar/gks216. 22402487 PMC3384332

[19] Swarts DC, Mosterd C, van Passel MWJ, Brouns SJJ. 2012. CRISPR interference directs strand specific spacer acquisition. PLoS One 7:e35888. doi:10.1371/journal.pone.0035888. 22558257 PMC3338789

[20] Datsenko KA, Pougach K, Tikhonov A, Wanner BL, Severinov K, Semenova E. 2012. Molecular memory of prior infections activates the CRISPR/Cas adaptive bacterial immunity system. Nat Commun 3:945–947. doi:10.1038/ncomms1937. 22781758

[21] Richter C, Dy RL, McKenzie RE, Watson BNJ, Taylor C, Chang JT, McNeil MB, Staals RHJ, Fineran PC. 2014. Priming in the type I-F CRISPR-Cas system triggers strand-independent spacer acquisition, bi-directionally from the primed protospacer. Nucleic Acids Res 42:8516–8526. doi:10.1093/nar/gku527. 24990370 PMC4117759

[22] Staals RHJ, Jackson SA, Biswas A, Brouns SJJ, Brown CM, Fineran PC. 2016. Interference-driven spacer acquisition is dominant over naive and primed adaptation in a native CRISPR-Cas system. Nat Commun 7:12853. doi:10.1038/ncomms12853. 27694798 PMC5059440

[23] Nicholson TJ, Jackson SA, Croft BI, Staals RHJ, Fineran PC, Brown CM. 2019. Bioinformatic evidence of widespread priming in type I and II CRISPR-Cas systems. RNA Biol 16:566–576. doi:10.1080/15476286.2018.1509662. 30157725 PMC6546363

[24] Nussenzweig PM, McGinn J, Marraffini LA. 2019. Cas9 cleavage of viral genomes primes the acquisition of new immunological memories. Cell Host Microbe 26:515–526.e6. doi:10.1016/j.chom.2019.09.002. 31585845 PMC7558852

[25] Modell JW, Jiang W, Marraffini LA. 2017. CRISPR-Cas systems exploit viral DNA injection to establish and maintain adaptive immunity. Nature 544:101–104. doi:10.1038/nature21719. 28355179 PMC5540373

[26] Wei Y, Chesne MT, Terns RM, Terns MP. 2015. Sequences spanning the leader-repeat junction mediate CRISPR adaptation to phage in *Streptococcus thermophilus*. Nucleic Acids Res 43:1749–1758. doi:10.1093/nar/gku1407. 25589547 PMC4330368

[27] Díez-Villaseñor C, Guzmán NM, Almendros C, García-Martínez J, Mojica FJM. 2013. CRISPR-spacer integration reporter plasmids reveal distinct genuine acquisition specificities among CRISPR-Cas I-E variants of *Escherichia coli*. RNA Biol 10:792–802. doi:10.4161/rna.24023. 23445770 PMC3737337

[28] Kieper SN, Almendros C, Brouns SJJ. 2019. Conserved motifs in the CRISPR leader sequence control spacer acquisition levels in type I-D CRISPR-Cas systems. FEMS Microbiol Lett 366:fnz129. doi:10.1093/femsle/fnz129. 31252430 PMC6607411

[29] Shah SA, Garrett RA. 2011. CRISPR/Cas and Cmr modules, mobility and evolution of adaptive immune systems. Res Microbiol 162:27–38. doi:10.1016/j.resmic.2010.09.001. 20863886

[30] Lillestøl RK, Shah SA, Brügger K, Redder P, Phan H, Christiansen J, Garrett RA. 2009. CRISPR families of the crenarchaeal genus *Sulfolobus*: bidirectional transcription and dynamic properties. Mol Microbiol 72:259–272. doi:10.1111/j.1365-2958.2009.06641.x. 19239620

[31] Garrett RA, Vestergaard G, Shah SA. 2011. Archaeal CRISPR-based immune systems: exchangeable functional modules. Trends Microbiol 19:549–556. doi:10.1016/j.tim.2011.08.002. 21945420

[32] Alkhnbashi OS, Shah SA, Garrett RA, Saunders SJ, Costa F, Backofen R. 2016. Characterizing leader sequences of CRISPR loci. Bioinformatics 32:i576–i585. doi:10.1093/bioinformatics/btw454. 27587677

[33] Makarova KS, Haft DH, Barrangou R, Brouns SJJ, Charpentier E, Horvath P, Moineau S, Mojica FJM, Wolf YI, Yakunin AF, van der Oost J, Koonin EV. 2011. Evolution and classification of the CRISPR-Cas systems. Nat Rev Microbiol 9:467–477. doi:10.1038/nrmicro2577. 21552286 PMC3380444

[34] Laanto E, Hoikkala V, Ravantti J, Sundberg L-R. 2017. Long-term genomic coevolution of host-parasite interaction in the natural environment. Nat Commun 8:111. doi:10.1038/s41467-017-00158-7. 28740072 PMC5524643

[35] Erdmann S, Garrett RA. 2012. Selective and hyperactive uptake of foreign DNA by adaptive immune systems of an archaeon via two distinct mechanisms. Mol Microbiol 85:1044–1056. doi:10.1111/j.1365-2958.2012.08171.x. 22834906 PMC3468723

[36] Pinilla-Redondo R, Mayo-Muñoz D, Russel J, Garrett RA, Randau L, Sørensen SJ, Shah SA. 2020. Type IV CRISPR-Cas systems are highly diverse and involved in competition between plasmids. Nucleic Acids Res 48:2000–2012. doi:10.1093/nar/gkz1197. 31879772 PMC7038947

[37] Shmakov S, Abudayyeh OO, Makarova KS, Wolf YI, Gootenberg JS, Semenova E, Minakhin L, Joung J, Konermann S, Severinov K, Zhang F, Koonin EV. 2015. Discovery and functional characterization of diverse class 2 CRISPR-Cas systems. Mol Cell 60:385–397. doi:10.1016/j.molcel.2015.10.008. 26593719 PMC4660269

[38] Sternberg SH, Richter H, Charpentier E, Qimron U. 2016. Adaptation in CRISPR-Cas systems. Mol Cell 61:797–808. doi:10.1016/j.molcel.2016.01.030. 26949040

[39] Smargon AA, Cox DBT, Pyzocha NK, Zheng K, Slaymaker IM, Gootenberg JS, Abudayyeh OA, Essletzbichler P, Shmakov S, Makarova KS, Koonin EV, Zhang F. 2017. Cas13b is a type VI-B CRISPR-associated RNA-guided RNase differentially regulated by accessory proteins Csx27 and Csx28. Mol Cell 65:618–630.e7. doi:10.1016/j.molcel.2016.12.023. 28065598 PMC5432119

[40] Barrangou R, Gersbach CA. 2017. Expanding the CRISPR toolbox: targeting RNA with Cas13b. Mol Cell 65:582–584. doi:10.1016/j.molcel.2017.02.002. 28212745

[41] Zhu Y, Klompe SE, Vlot M, van der Oost J, Staals RHJ. 2018. Shooting the messenger: RNA-targeting CRISPR-Cas systems. Biosci Rep 38:BSR20170788. doi:10.1042/BSR20170788. 29748239 PMC6013697

[42] Deng L, Garrett RA, Shah SA, Peng X, She Q. 2013. A novel interference mechanism by a type IIIB CRISPR‐Cmr module in *Sulfolobus*. Mol Microbiol 87:1088–1099. doi:10.1111/mmi.12152. 23320564

[43] Silas S, Lucas-Elio P, Jackson SA, Aroca-Crevillén A, Hansen LL, Fineran PC, Fire AZ, Sánchez-Amat A. 2017. Type III CRISPR-Cas systems can provide redundancy to counteract viral escape from type I systems. Elife 6:e27601. doi:10.7554/eLife.27601. 28826484 PMC5576922

[44] Dugar G, Leenay RT, Eisenbart SK, Bischler T, Aul BU, Beisel CL, Sharma CM. 2018. CRISPR RNA-dependent binding and cleavage of endogenous RNAs by the *Campylobacter jejuni* Cas9. Mol Cell 69:893–905.e7. doi:10.1016/j.molcel.2018.01.032. 29499139 PMC5859949

[45] Rousseau BA, Hou Z, Gramelspacher MJ, Zhang Y. 2018. Programmable RNA cleavage and recognition by a natural CRISPR-Cas9 system from *Neisseria meningitidis*. Mol Cell 69:906–914.e4. doi:10.1016/j.molcel.2018.01.025. 29456189 PMC5889306

[46] Maikova A, Peltier J, Boudry P, Hajnsdorf E, Kint N, Monot M, Poquet I, Martin-Verstraete I, Dupuy B, Soutourina O. 2018. Discovery of new type I toxin-antitoxin systems adjacent to CRISPR arrays in *Clostridium difficile*. Nucleic Acids Res 46:4733–4751. doi:10.1093/nar/gky124. 29529286 PMC5961336

[47] Koonin EV, Zhang F. 2017. Coupling immunity and programmed cell suicide in prokaryotes: life‐or‐death choices. Bioessays 39:1–9. doi:10.1002/bies.201600186. 27896818

[48] Zhang Y, Heidrich N, Ampattu BJ, Gunderson CW, Seifert HS, Schoen C, Vogel J, Sontheimer EJ. 2013. Processing-independent CRISPR RNAs limit natural transformation in *Neisseria meningitidis*. Mol Cell 50:488–503. doi:10.1016/j.molcel.2013.05.001. 23706818 PMC3694421

[49] Meeske AJ, Nakandakari-Higa S, Marraffini LA. 2019. Cas13-induced cellular dormancy prevents the rise of CRISPR-resistant bacteriophage. Nature 570:241–245. doi:10.1038/s41586-019-1257-5. 31142834 PMC6570424

[50] Ravantti JJ, Laanto E, Papponen P, Sundberg L-R. 2019. Complete genome sequence of fish pathogen *Flavobacterium columnare* strain B185, originating from Finland. Microbiol Resour Announc 8:e01285-19. doi:10.1128/MRA.01285-19. 31806749 PMC6895309

[51] Laanto E, Sundberg L-R, Bamford JKH. 2011. Phage specificity of the freshwater fish pathogen *Flavobacterium columnare*. Appl Environ Microbiol 77:7868–7872. doi:10.1128/AEM.05574-11. 21890667 PMC3209183

[52] Hynes AP, Villion M, Moineau S. 2014. Adaptation in bacterial CRISPR-Cas immunity can be driven by defective phages. Nat Commun 5:4399. doi:10.1038/ncomms5399. 25056268

[53] Stern A, Keren L, Wurtzel O, Amitai G, Sorek R. 2010. Self-targeting by CRISPR: gene regulation or autoimmunity? Trends Genet 26:335–340. doi:10.1016/j.tig.2010.05.008. 20598393 PMC2910793

[54] Staroscik AM, Hunnicutt DW, Archibald KE, Nelson DR. 2008. Development of methods for the genetic manipulation of *Flavobacterium columnare*. BMC Microbiol 8:115. doi:10.1186/1471-2180-8-115. 18620586 PMC2483708

[55] Rauch BJ, Silvis MR, Hultquist JF, Waters CS, McGregor MJ, Krogan NJ, Bondy-Denomy J. 2017. Inhibition of CRISPR-Cas9 with bacteriophage proteins. Cell 168:150–158.e10. doi:10.1016/j.cell.2016.12.009. 28041849 PMC5235966

[56] Leenay RT, Beisel CL. 2017. Deciphering, communicating, and engineering the CRISPR PAM. J Mol Biol 429:177–191. doi:10.1016/j.jmb.2016.11.024. 27916599 PMC5235977

[57] Chen S, Bagdasarian M, Kaufman MG, Walker ED. 2007. Characterization of strong promoters from an environmental *Flavobacterium hibernum* strain by using a green fluorescent protein-based reporter system. Appl Environ Microbiol 73:1089–1100. doi:10.1128/AEM.01577-06. 17189449 PMC1828668

[58] Lopatina A, Medvedeva S, Shmakov S, Logacheva MD, Krylenkov V, Severinov K. 2016. Metagenomic analysis of bacterial communities of Antarctic surface snow. Front Microbiol 7:398. doi:10.3389/fmicb.2016.00398. 27064693 PMC4814470

[59] Laanto E, Bamford JKH, Ravantti JJ, Sundberg L-R. 2015. The use of phage FCL-2 as an alternative to chemotherapy against columnaris disease in aquaculture. Front Microbiol 6:829. doi:10.3389/fmicb.2015.00829. 26347722 PMC4541368

[60] Levy A, Goren MG, Yosef I, Auster O, Manor M, Amitai G, Edgar R, Qimron U, Sorek R. 2015. CRISPR adaptation biases explain preference for acquisition of foreign DNA. Nature 520:505–510. doi:10.1038/nature14302. 25874675 PMC4561520

[61] Goren MG, Doron S, Globus R, Amitai G, Sorek R, Qimron U. 2016. Repeat size determination by two molecular rulers in the type I-E CRISPR array. Cell Rep 16:2811–2818. doi:10.1016/j.celrep.2016.08.043. 27626652 PMC5039180

[62] He Y, Wang M, Liu M, Huang L, Liu C, Zhang X, Yi H, Cheng A, Zhu D, Yang Q, Wu Y, Zhao X, Chen S, Jia R, Zhang S, Liu Y, Yu Y, Zhang L. 2018. Cas1 and Cas2 from the type II-C CRISPR-Cas system of *Riemerella anatipestifer* are required for spacer acquisition. Front Cell Infect Microbiol 8:195. doi:10.3389/fcimb.2018.00195. 29951376 PMC6008519

[63] Wang J, Li J, Zhao H, Sheng G, Wang M, Yin M, Wang Y. 2015. Structural and mechanistic basis of PAM-dependent spacer acquisition in CRISPR-Cas systems. Cell 163:840–853. doi:10.1016/j.cell.2015.10.008. 26478180

[64] Nuñez JK, Kranzusch PJ, Noeske J, Wright AV, Davies CW, Doudna JA. 2014. Cas1-Cas2 complex formation mediates spacer acquisition during CRISPR-Cas adaptive immunity. Nat Struct Mol Biol 21:528–534. doi:10.1038/nsmb.2820. 24793649 PMC4075942

[65] Rollie C, Schneider S, Brinkmann AS, Bolt EL, White MF. 2015. Intrinsic sequence specificity of the Cas1 integrase directs new spacer acquisition. Elife 4:e08716. doi:10.7554/eLife.08716. PMC4574026 26284603

[66] Arslan Z, Hermanns V, Wurm R, Wagner R, Pul Ü. 2014. Detection and characterization of spacer integration intermediates in type I-E CRISPR-Cas system. Nucleic Acids Res 42:7884–7893. doi:10.1093/nar/gku510. 24920831 PMC4081107

[67] Shmakov S, Savitskaya E, Semenova E, Logacheva MD, Datsenko KA, Severinov K. 2014. Pervasive generation of oppositely oriented spacers during CRISPR adaptation. Nucleic Acids Res 42:5907–5916. doi:10.1093/nar/gku226. 24728991 PMC4027179

[68] Jackson SA, McKenzie RE, Fagerlund RD, Kieper SN, Fineran PC, Brouns SJJ. 2017. CRISPR-Cas: adapting to change. Science 356:eaal5056. doi:10.1126/science.aal5056. 28385959

[69] Chylinski K, Makarova KS, Charpentier E, Koonin EV. 2014. Classification and evolution of type II CRISPR-Cas systems. Nucleic Acids Res 42:6091–6105. doi:10.1093/nar/gku241. 24728998 PMC4041416

[70] Hynes AP, Lemay M-L, Trudel L, Deveau H, Frenette M, Tremblay DM, Moineau S. 2017. Detecting natural adaptation of the *Streptococcus thermophilus* CRISPR-Cas systems in research and classroom settings. Nat Protoc 12:547–565. doi:10.1038/nprot.2016.186. 28207002

[71] Pawluk A, Davidson AR, Maxwell KL. 2018. Anti-CRISPR: discovery, mechanism and function. Nat Rev Microbiol 16:12–17. doi:10.1038/nrmicro.2017.120. 29062071

[72] Shieh HS. 1980. Studies on the nutrition of a fish pathogen, *Flexibacter columnaris*. Microbios Lett 13:129–133.

[73] Mäki A, Rissanen AJ, Tiirola M. 2016. A practical method for barcoding and size-trimming PCR templates for amplicon sequencing. Biotechniques 60:88–90. doi:10.2144/000114380. 26842354

[74] Bolger AM, Lohse M, Usadel B. 2014. Trimmomatic: a flexible trimmer for Illumina sequence data. Bioinformatics 30:2114–2120. doi:10.1093/bioinformatics/btu170. 24695404 PMC4103590

[75] Fu L, Niu B, Zhu Z, Wu S, Li W. 2012. CD-HIT: accelerated for clustering the next-generation sequencing data. Bioinformatics 28:3150–3152. doi:10.1093/bioinformatics/bts565. 23060610 PMC3516142

[76] Langmead B, Salzberg SL. 2012. Fast gapped-read alignment with Bowtie 2. Nat Methods 9:357–359. doi:10.1038/nmeth.1923. 22388286 PMC3322381

[77] Luo H, Gao F. 2019. DoriC 10.0: an updated database of replication origins in prokaryotic genomes including chromosomes and plasmids. Nucleic Acids Res 47:D74–D77. doi:10.1093/nar/gky1014. 30364951 PMC6323995

[78] Crooks GE, Hon G, Chandonia J-M, Brenner SE. 2004. WebLogo: a sequence logo generator. Genome Res 14:1188–1190. doi:10.1101/gr.849004. 15173120 PMC419797

[79] Li N, Qin T, Zhang XL, Huang B, Liu ZX, Xie HX, Zhang J, McBride MJ, Nie P. 2015. Gene deletion strategy to examine the involvement of the two chondroitin lyases in *Flavobacterium columnare* virulence. Appl Environ Microbiol 81:7394–7402. doi:10.1128/AEM.01586-15. 26253667 PMC4592881

[80] Farmer BD. 2004. Improved methods for the isolation and characterization of *Flavobacterium columnare*. Master’s thesis. Department of Pathobiological Sciences, Louisiana State University, Baton Rouge, LA.

[81] Couvin D, Bernheim A, Toffano-Nioche C, Touchon M, Michalik J, Néron B, Rocha EPC, Vergnaud G, Gautheret D, Pourcel C. 2018. CRISPRCasFinder, an update of CRISRFinder, includes a portable version, enhanced performance and integrates search for Cas proteins. Nucleic Acids Res 46:W246–W251. doi:10.1093/nar/gky425. 29790974 PMC6030898

