# Supplementary material for: Cooperation between Different CRISPR-Cas Types Enables Adaptation in an RNA-Targeting System
Source: mBio. 2021 Mar 30;12(2):e03338-20. doi: 10.1128/mBio.03338-20 (PMC8092290; doi:10.1128/mBio.03338-20)
Supplement: TABLE S1 [file mBio.03338-20-st001.docx]

A

| Condition^a^ | Replicate | Screened | Number of mutants in initial screen^B^ | Number of mutants after serial plating^C^ |
| --- | --- | --- | --- | --- |
| B + P + UVP | a | 15 | 5 | 1 |
| B + P + UVP | b | 3 | 0 | 0 |
| B + P + UVP | c | 40 | 8 | 4 |
| B + P + UVP | d | 14 | 1 | 1 |
| B + P + UVP | e | 13 | 0 | 0 |
| B+ P | a | 20 | 0 | 0 |
| B+ P | b | 0 | 0 | 0 |
| B+ P | c | 20 | 0 | 0 |
| B+ P | d | 21 | 0 | 0 |
| B+ P | e | 19 | 0 | 0 |
| B + UVP | a | 20 | 0 | 0 |
| B + UVP | b | 20 | 0 | 0 |
| B + UVP | c | 7 | 0 | 0 |
| B + UVP | d | 10 | 0 | 0 |
| B + UVP | e | 20 | 0 | 0 |
| B | a | 4 | 0 | 0 |
| B | b | 4 | 0 | 0 |
| B | c | 7 | 0 | 0 |
| B | d | 7 | 0 | 0 |
| B | e | 17 | 0 | 0 |

B

| Mutant ID | Locus | Morphotype | Spacer target | Sequence |
| --- | --- | --- | --- | --- |
| 1c30z | II-C | Rhizoid | FCL2 (ORF64) | AAGTGCTAACTTCAATACGCTGCATGGTGG |
| 1a5z | VI-B | Rhizoid | Self (RHS repeat-associated core domain protein) | CAACTTGTCATTCTCATCAAAAGGCCCTTC |
| 1c13z | VI-B | Rhizoid | Self (diacylglyceryl transferase) | TAAGATGTCCTTTTTAATTATTTTTCAGAG |
| 1c38r | VI-B | Rough | FCL2 (ORF58) | AACGATTTAGCGTTAATAATTTCGTTCGA |
| 1d14z | VI-B | Rhizoid | FCL2 (ORF77) (1bp over ORF) | TTCTGCCCTCTAAATTTAATATCTCTCATA |
| 1c5z | 1x II-C,  2x VI-B | Rhizoid | II-C: FCL2 (ORF58)  VI-B: FCL2 (ORF64) & self (damage-inducible protein) | AAATTTGTTTACAATTTGAACTTGTTCCAG,  TAAAATTTTATTGTTTTTAAACCGCTTTC, TTACCGGGAGTGCCTTATGAAATGAAACAC |
