# Supplementary material for: Cooperation between Different CRISPR-Cas Types Enables Adaptation in an RNA-Targeting System
Source: mBio. 2021 Mar 30;12(2):e03338-20. doi: 10.1128/mBio.03338-20 (PMC8092290; doi:10.1128/mBio.03338-20)
Supplement: TABLE S2 [file mBio.03338-20-st002.docx]

A

| Primer | Purpose | Sequence |
| --- | --- | --- |
| C2_B185_F | Type VI-B array, B185 specific | ACTATGAGAGGCTTACCAGCGTTTA |
| C2_B185_R | Type VI-B array, B185 specific | CAAACACATTGTATCATTTAGCAGTAA |
| C1_B185_F | Type II-C array, B185 specific | TGTTCTATCGGCGTAAAATAAGTT |
| C1_B185_R | Type II-C array, B185 specific | TAACGATATTTCGGCTTAATGCT |
| M13-B185_223bp_C2F | Type VI-B, B185, M13 underlined | TGTAAAACGACGGCCAGTACTATGAGAGGCTTACCAGCGTTTA |
| P1-B185_223bp_C2R | Type VI-B, B185, P1 underlined | CCTCTCTATGGGCAGTCGGTGATCAAACACATTGTATCATTTAGCAGTAA |
| P1-B185_181bp_C1R | Type II-C, B185, P1 underlined | CCTCTCTATGGGCAGTCGGTGATTAACGATATTTCGGCTTAATGCT |
| M13-B185_C1_F3 | Type II-C, B185, M13 underlined | TGTAAAACGACGGCCAGTTGTTCTATCGGCGTAAAATAAGTT |
| B245_C1_F | Type II-C, B245 specific | CTGTTTTGTTTCATTTGGTAAATCA |
| B245_C2_R | Type VI-B, B245 specific | GATGTAGAAATACTTAGCGACAATGTAG |
| F_col_C2_F | Type VI-B, *F. columnare* common | GGTCTAAATACAATTGCTCTTTGACATT |
| F_col_C1_R | Type II-C, *F. columnare* common | CCCTAAAGCACCACAACCCA |
| B245_cas1_F | Cas1 sequencing primer (B245) | TGTACATCGGCACTTTCGCT |
| B245_cas1_R | Cas1 sequencing primer (B245) | AGTATTCCCGCCCCGTATTT |
| 2322 | B245 cas1 upstream fragment F | GCTAGGGTACCAACGGATGGAGCAATAAGTGT |
| 2323 | B245 cas1 upstream fragment R | GCTAGGGATCCAGTAGCCTCTCGTAAAATCCC |
| 2324 | B245 cas1 downstream fragment F | GCTAGGGATCCGCAAGTTCCTTACAACAGTGT |
| 2325 | B245 cas1 downstream fragment R | GCTAGGTCGACAGCTAACAGCTTAGTTATTAAATATCAAAG |
| 2367 | Plasmid sequencing primer | GCTAGGGTACCAATTCGATACCCGTTTAAATCCA |
| 2368 | Plasmid sequencing primer | GCTAGGGATCCCTAAAAGAGCTGTTATATTCATCG |
| pAS43_protoSDM_F | Matching protospacer insertion oligo F (lowercase = insertion, uppercase = plasmid-complementary, underlined = PAM) | gtatgtacgaactgctaaaCCCGGGAATTCAGCAATAG |
| pAS43_protoSDM_R | Matching protospacer insertion oligo R (lowercase = insertion, uppercase = plasmid-complementary) | tcatttgttttaaaattaccTCGACGGTATCGATAAGC |
| pAS43_protoSDM_neg_F | Non-matching protospacer insertion oligo F (lowercase = insertion, uppercase = plasmid-complementary, underlined = PAM) | caatgtatgaactgctaaaCCCGGGAATTCAGCAATAG |
| pAS43_protoSDM_neg_R | Non-matching protospacer insertion oligo R (lowercase = insertion, uppercase = plasmid-complementary) | acatagagattagatctgatTCGACGGTATCGATAAGC |
| pAS43_MCS2_seq_F | Protospacer insertion sequencing primer F | TCGGCTTGAACCATCTGTCC |
| pAS43_MCS2_seq_R | Protospacer insertion sequencing primer R | GGCAGGTCGCACGAGATTAT |

B

| Sample | Locus | Number of reads | Total spacers after filtering | Unique spacers |
| --- | --- | --- | --- | --- |
| B + P d | II-C | 71249 | 12630 | 646 |
| B + P e | II-C | 71577 | 11438 | 569 |
| B + P b | VI-B | 69081 | 1825 | 474 |
| B + P d | VI-B | 56903 | 768 | 331 |
| B + P e | VI-B | 57828 | 1046 | 418 |
