# Supplementary material for: Cooperation between Different CRISPR-Cas Types Enables Adaptation in an RNA-Targeting System
Source: mBio. 2021 Mar 30;12(2):e03338-20. doi: 10.1128/mBio.03338-20 (PMC8092290; doi:10.1128/mBio.03338-20)
Supplement: TABLE S3 [file mBio.03338-20-st003.docx]

A

| ARRAY POSITIoN | II-C CRRNA | Ii-C Target | target STRAND | TARGET POSITION | PAM (downstream) |
| --- | --- | --- | --- | --- | --- |
| 1 | UUAAUUAAAUCACAAAAAAUGACAAGAGA | FCL-2 | - | 12966-12994 | GGAATTAAAA |
| 2 | AUGUUCUAUCGGCGUAAAAUAAGUUAGCAG | FC-L2 | + | 40131-40160 | TTAGATAAAA |
| 3 | UACUCAUUCUCACUAAAAGAAAUAGCUUA |  |  |  |  |
| 4 | GUUAUUCAAGAUAAUUGCAACAUAGGGUA |  |  |  |  |
| 5 | CGACCGCAAACAUACGAUAGUUACUUUAUA | FCL-2* | - | 42621-42644 | AAAAGTAAAG |
| 6 | GGUUAUAAAAUUGAUGAAGAAAUAGGCUA |  |  |  |  |
| 7 | AGAAUUUCGGUUGGAUACUCAAAGCAAUUG |  |  |  |  |
| 8 | UUAUAACCUUUUUACUAUUUCAUUUAGCUU |  |  |  |  |
| 9 | GAUGCUGAAGAAAAACACAAAAAAGAACAA |  |  |  |  |
| 10 | CUUAUUUUUCAAGUCCGUCGGGGUCGAUG |  |  |  |  |
| 11 | AACUUGCAAACAAUACCAGACUAUCAAAAA |  |  |  |  |
| 12 | GCAUUUCAAGAUAGUAGAGAAUUUAAUCAG | FCL-2 | - | 23720-23749 | CCCAATAAAA |
| 13 | AAAAUAAGAAGAGAUAGUAACAGAAACAAA |  |  |  |  |
| 14 | UCUACAAAUACCUGAAUUAACUCUAAACUG |  |  |  |  |
| 15 | UUUGAAAUAAUUUUUAAUUUUAUAACAUUA |  |  |  |  |
| 16 | GAAAGCUAUGCUGCCAUAUAAACAGGAUGG |  |  |  |  |
| 17 | UAUAAAGCAAAACUAAUUGAUUGAUAAACU |  |  |  |  |
| 18 | UUCAGAAGGAAUUUAGAAGCUUCUGUUCCU |  |  |  |  |
| 19 | AUUAUAUUUGUGUUACAUGGUGUAGUUAAA |  |  |  |  |
| 20 | AUUGUAAGGUAUUUCAAGUGGACACCGCA | FCL-2 | - | 18513-18541 | AACATTAAAT |
| 21 | UUUUAAAUUUGGCGGUUGACCAUAGAUCG |  |  |  |  |
| 22 | CGAUUUGGUUACACGAAGGUAUUUUCGAG |  |  |  |  |
| 23 | UGACUAAAAAAGCCAUUGCAAGCGCUACAG |  |  |  |  |
| 24 | AUUUUAAAAAGACAGAUUAAAUCGGCUGGG |  |  |  |  |

B

| ARRAY POSITION | VI-B crRNA sequence | VI-B Target | target Strand | Target position | PAM (upstream) |
| --- | --- | --- | --- | --- | --- |
| 1 | UAUUCUAAUGGAUCAAUUAAAAAAGCAAGA | self | - | 751992- 752021 | ATTTATTCCC |
| 2 | UCGAGAGGUGUUAAUGAAACGAACAUAGUU | self | - | 1885766- 1885737 | TTTTACTTCC |
| 3 | AAUUACUGCUAAAUGAUACAAUGUGUUUG |  |  |  |  |
| 4 | GGUAAAUAAUUUAUCUCUAGAUUAUAGUAU | self | - | 2006885- 2006914 | ATTTACTGCT |
| 5 | GCUAUGCUACUUAUACAGAUGCACAACAUC | self | - | 1936639- 1936610 | TTTTATGGCA |
| 6 | UGGUUUUCCGUAAUGAUAUAAAUAGAAUUU |  |  |  |  |
| 7 | CGGUUUGUCUGCAUUUGAAACUCCAGCAC | self | - | 2730639- 2730667 | CTTTAATATT |
| 8 | UCGCGGGAAAAGUUGAAAAAGGAUUAAAC |  |  |  |  |
| 9 | GUUUUAACCAAACAUUUGCCAGGGCAUAA | self | - | 2626284- 2626312 | TTTAAAACAA |
| 10 | AGUGCGGAAAGCCCGGCUUUAGAACGCCCA |  |  |  |  |
| 11 | GGAGACAUCCAUCCUCUAUCUAAACGUAAA |  |  |  |  |
| 12 | AAUGAAGAAGUUUAUUUAGAAAACCAAAGA |  |  |  |  |
| 13 | CAAUCUCAAAGUAUAAUUGAUAAAUAGGC | FCL2* | + | 8728-8756 | TTTAAAACTT |
| 14 | UUCUAGCUUUGGCCACCUCAUCUUUUGAA | FCL2* | + | 44354-44382 | TTTAATACTT |
| 15 | UUGUCGAACUUUUCUUUUUUAUAAUGCAA | FCL2* | + | 40064-40092 | TTTACCTTCT |
| 16 | CAUAUUCUUCUUCAAAGUGAUGUUUGUAAU | FCL2* | + | 42775-42804 | ATATAATCAT |

C

| ARRAY POSITIoN | II-C crRNA sequence | II-C Target | target Strand | Target position | PAM (downstream) |
| --- | --- | --- | --- | --- | --- |
| 1 | GGUAAUUUUAAAACAAAUGAGUAUGUACGA | V156 | - | 23543-23572 | TTAATTAAAA |
| 2 | UACUGUUUUGUUUCAUUUGGUAAAUCAAGA | V156 | - | 6008-6037 | AGCTATAAAA |
| 3 | UUGUAAAUUUUUAAAAACUUCUAAAAGAGA | V156 | - | 37104-37133 | TGTTTTAAAA |
| 4 | UCGUAUCAUUUGACAUCUAAGAUAAUAUAU | V156* | - | 39968-39991 | GAAACAAAGA |
| 5 | UUAAGUCAUGAUACCGAUGGAUUAUCGGUA | V156 | - | 15212-15241 | AATGCTAAAA |
| 6 | CTAATGGATTATTCATTGACGTAAUUGAU | Self* | + (partly ig) | 368067-368090 | CAACGGGAGT |
| 7 | ACAAAUAGUUAAAUCCCAGCCGUUGUAUUG |  |  |  |  |
| 8 | AUAUAAAACUAAAAAUAAAUUUAUAGUAAU |  |  |  |  |
| 9 | UUAAAAAACAAAUUAAAUCUUGAAGACCA |  |  |  |  |
| 10 | UGGUCAAGCAGAUUCUGUAGAGAAACUUCU |  |  |  |  |
| 11 | CUUUUACAAUGCACGAAAUUGUAGAAAAUG |  |  |  |  |
| 12 | AAAACCAUAAGAAUUAUUUAAAAAGUUACC |  |  |  |  |
| 13 | CAAUUUUAAUUUAUGACUGAAUUUGAAAUG |  |  |  |  |
| 14 | AUAUCUUUUCUUUUACAGGAAACAAUAAGA |  |  |  |  |
| 15 | GAAAGAAGCUAAAGCGAUGUUAAGAGCAUU |  |  |  |  |
| 16 | UUAUUGCCGGAACACCAGCGAAUGAAGUAA |  |  |  |  |
| 17 | CAAAUCACAAUCAUUUGAGAUUGAAUAGUG |  |  |  |  |
| 18 | UCACGCUAAAAAUAAUUUUAACAUUGAUUU |  |  |  |  |
| 19 | AGAAUGUAGAGAUUUAUUAAAUGAAAUUGA |  |  |  |  |
| 20 | AUUUUUCAAUUCACCGUGGGUCGCCACCC |  |  |  |  |
| 21 | GAUAUAAAGAGGUUGUACAAUAAAACCGA |  |  |  |  |
| 22 | UAAGCCGUGGGGUUCGAUCCCGACACCGAA |  |  |  |  |
| 23 | GUAAACAAGUUUAUUAUACCUGGCACGAU |  |  |  |  |
| 24 | AUGGUGCUGAGUAAUUCAUUACUCAUAAAC |  |  |  |  |
| 25 | UCACGCUUUAAGAUUGCGUUCUUACGAAGC |  |  |  |  |
| 26 | UUAGUAAUCAAAAUUAAUUUUGAAUUGCAU |  |  |  |  |
| 27 | GCUGGUUUGAGAAACGAAGGCGAGGUCUUU |  |  |  |  |
| 28 | CGAGCUAAUUCUUCAGCUAAACGUUGUUUG |  |  |  |  |
| 29 | UUAUUUUGAAAAAAAUUCAAUUAUAGGGAU | V156* | - | 426-446 | TAGCGGGTAA |
| 30 | AAAAAUUUGUUAAAGUUUUGUUGAAAAGUA |  |  |  |  |
| 31 | UUUAGAGUGCAAUACGGAAAUUUCCGAGUA |  |  |  |  |
| 32 | UAAAUCAUCAGUAGUUCGUAUUGUGGAUAU |  |  |  |  |
| 33 | AAUGGUCGGACAACCUAUAUACACCUUCUU |  |  |  |  |
| 34 | UAAAUAAAAAAAGUUUAGUAAAAGUUUAG |  |  |  |  |
| 35 | ACAAAUAUAAAUUAUGACAACUUUAUCAUU |  |  |  |  |
| 36 | AUUUUAUUUUUUUCAAAGGUCAAGGAUGGA |  |  |  |  |
| 37 | UCAUCAUUUAUUAAUGAAUCAAAAUUAUCA |  |  |  |  |

D

| Array position | VI-B crRNA sequence | VI-B Target | target Strand | Target position | PAM (upstream) |
| --- | --- | --- | --- | --- | --- |
| 1 | CAAUAGCUUUGCUUAAUGUAUCAGAAUUA | V156 | + | 19740-19768 | TTTATACTTT |
| 2 | AACCCGACUGUAUUGAUAUUUUUUUGAUAA | V156 | + | 41980- 42009 | TTTTACCACT |
| 3 | UACAUUGUCGCUAAGUAUUUCUACAUCUC |  |  |  |  |
| 4 | UUGUAUUUAGUAGAACUUAAAAUAUACCUA | V156 | + | 15789- 15818 | ATTTAAGTAC |
| 5 | UUUUAUGUUUUUGACCGUAAUCAAAAGAUA |  |  |  |  |
| 6 | CGAUUAAUUUUAGAUAAACCAAGAACUUUG |  |  |  |  |
| 7 | AUGAUAAAUAAGUUUUGUAUGAUGAUGUA |  |  |  |  |
| 8 | AUUUCUUUUAGUCUUAUUCUUUUCUUGAA |  |  |  |  |
| 9 | CCUGCUGUAAUUGCUGUAAUUGCUGAAUA |  |  |  |  |
| 10 | AGUUAGAUUUAUAUCAAGUAUUUUAGCCUG |  |  |  |  |
| 11 | UCUAUUACAGUGUAAUAUUCUUCAGGUAUU |  |  |  |  |
| 12 | AUAAAAUCCAUUUUUUCAAAAUAAUUUAAA | B245 | + (partly ig) | 137878- 137903 | GCTTAATCGC |
| 13 | GCAUUUGAUAUUUUAUAAUUGUUCCGAUUU |  |  |  |  |
| 14 | UCUACUGUUUUUAUUAACUUGAUAAACAGU |  |  |  |  |
| 15 | GUUUUUUGAAUAAAUAGUUCAUAAUCGUAA |  |  |  |  |
| 16 | AUUUGAGAUUGGUGGAUUAGCCUUAUCAU |  |  |  |  |
| 17 | ACGCCUCUAUUUCACGUUUAUGGUAAAAA |  |  |  |  |
