# Supplementary figures and images for: Cooperation between Different CRISPR-Cas Types Enables Adaptation in an RNA-Targeting System
Source: mBio. 2021 Mar 30;12(2):e03338-20. doi: 10.1128/mBio.03338-20 (PMC8092290; doi:10.1128/mBio.03338-20)

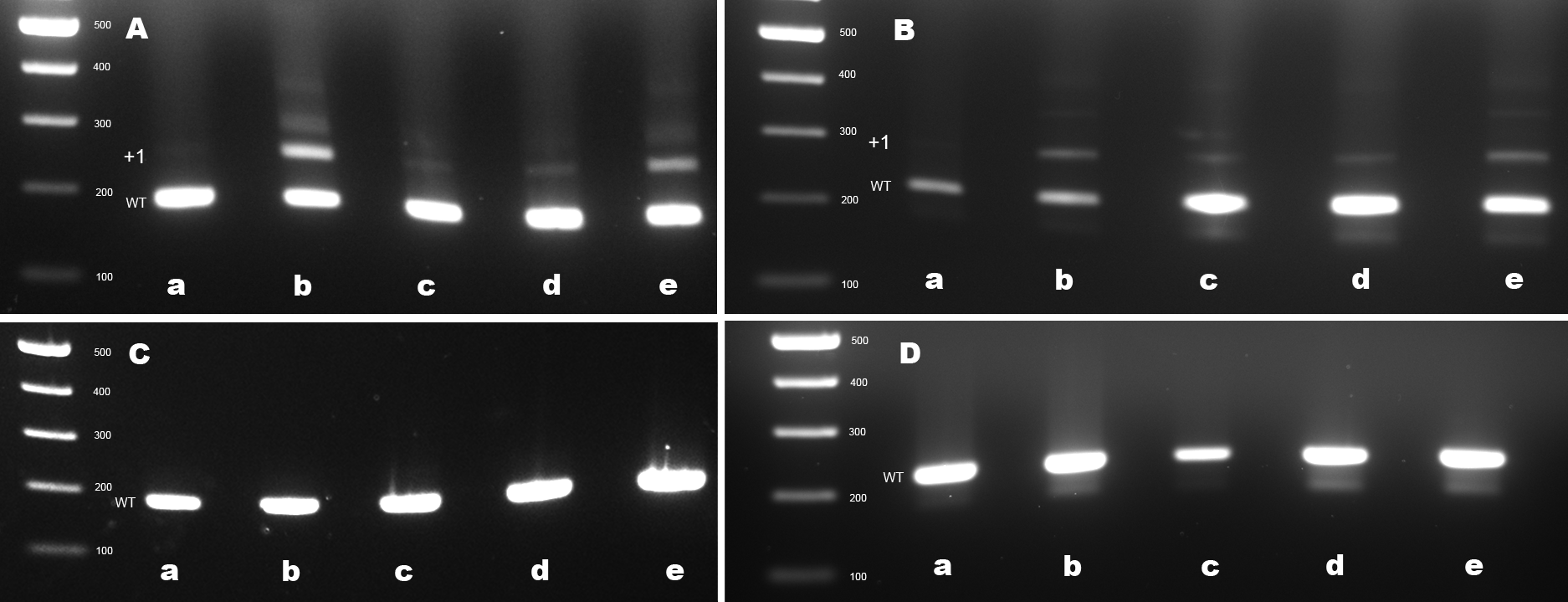

Supplement: FIG S1 [file mBio.03338-20-sf001.tif]

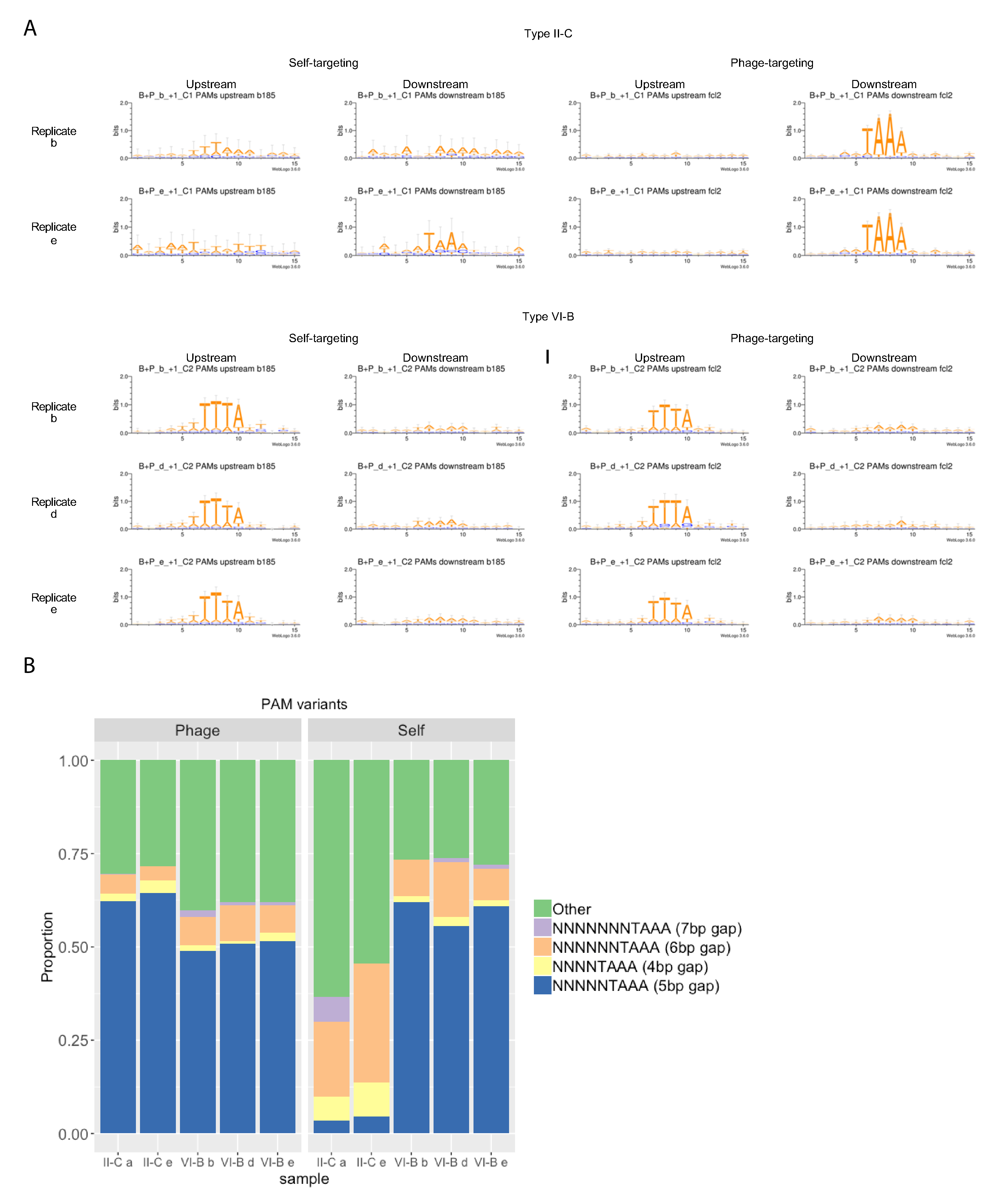

Supplement: FIG S2 [file mBio.03338-20-sf002.tif]

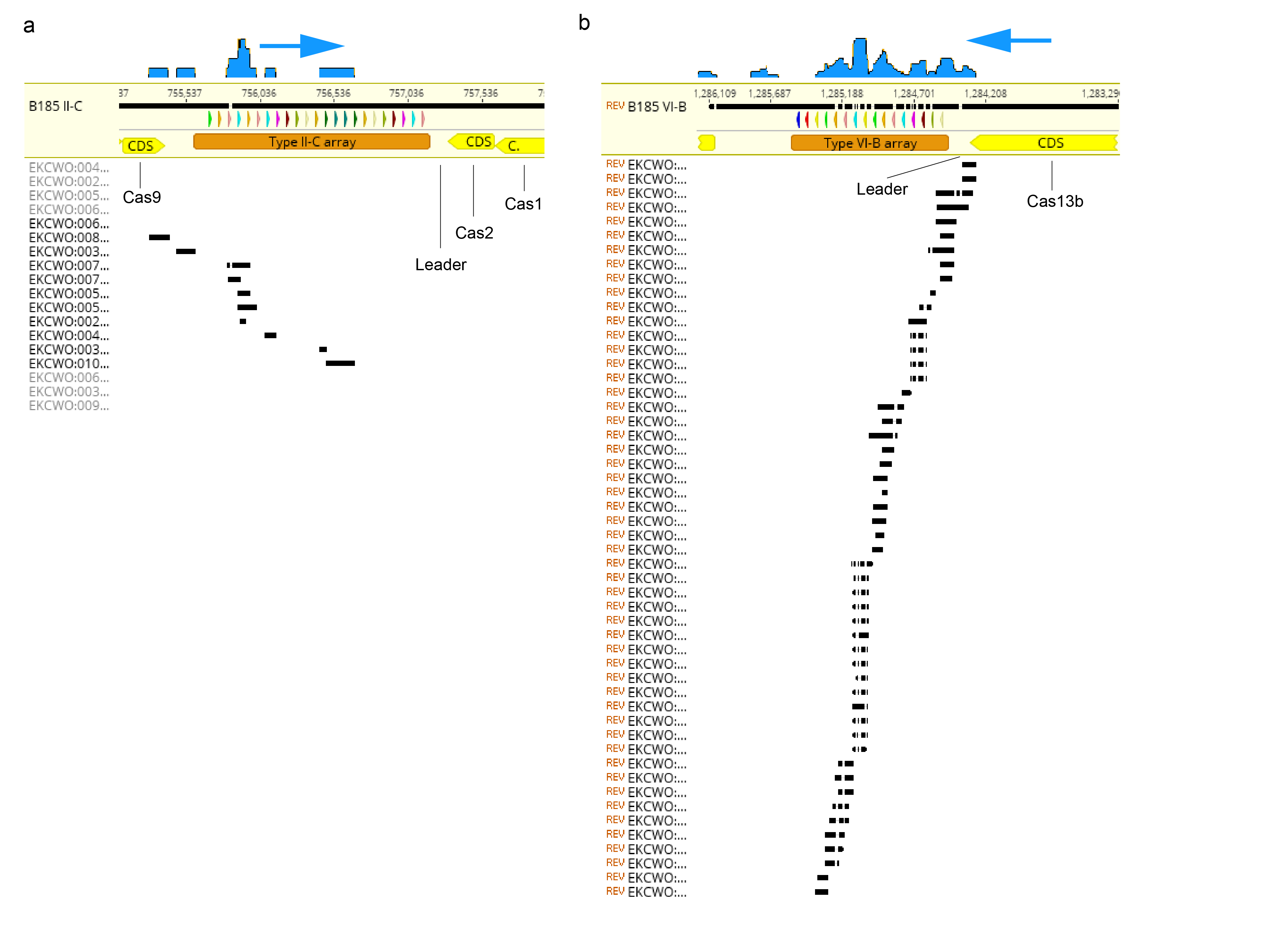

Supplement: FIG S3 [file mBio.03338-20-sf003.tif]

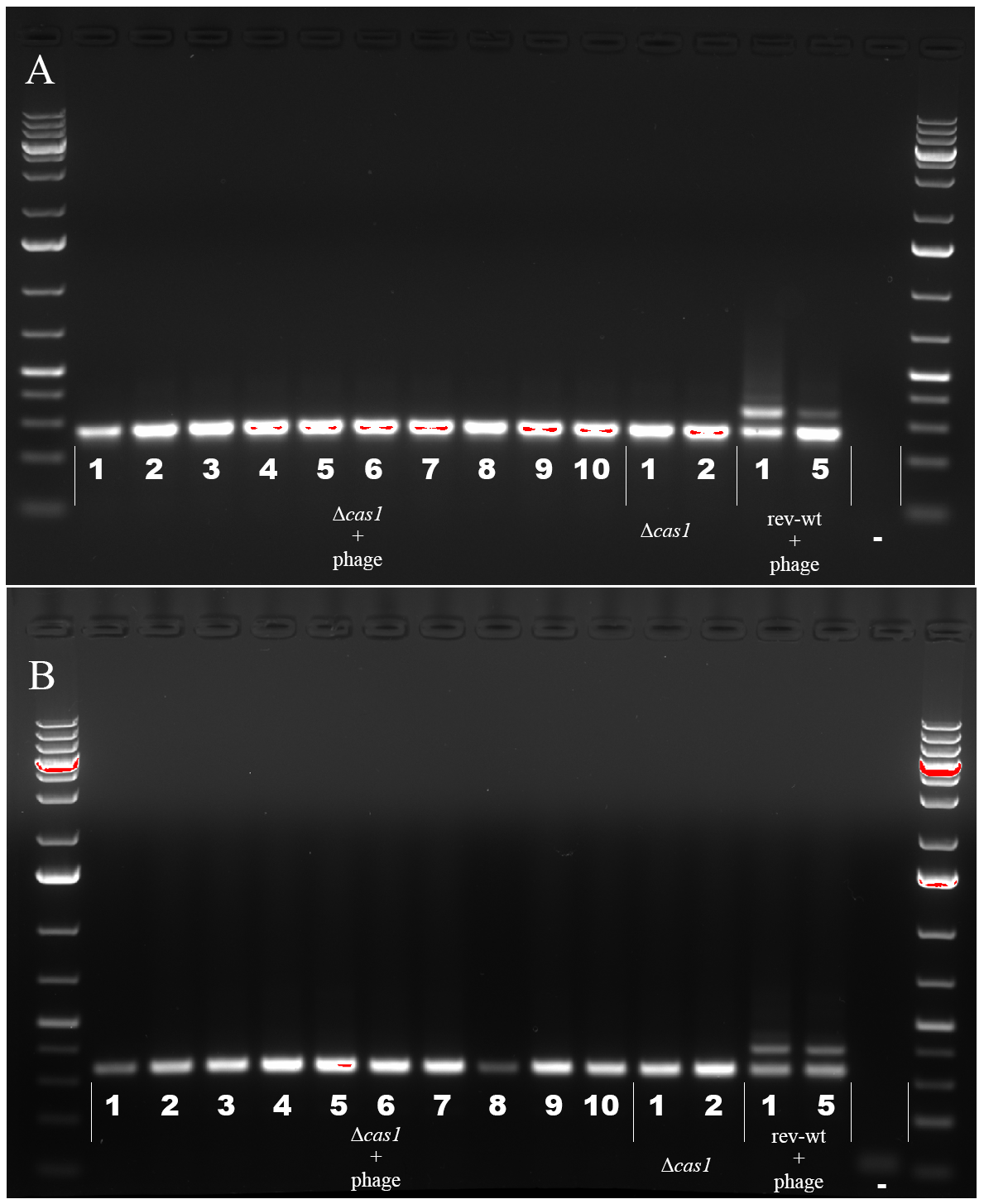

Supplement: FIG S4 [file mBio.03338-20-sf004.tif]

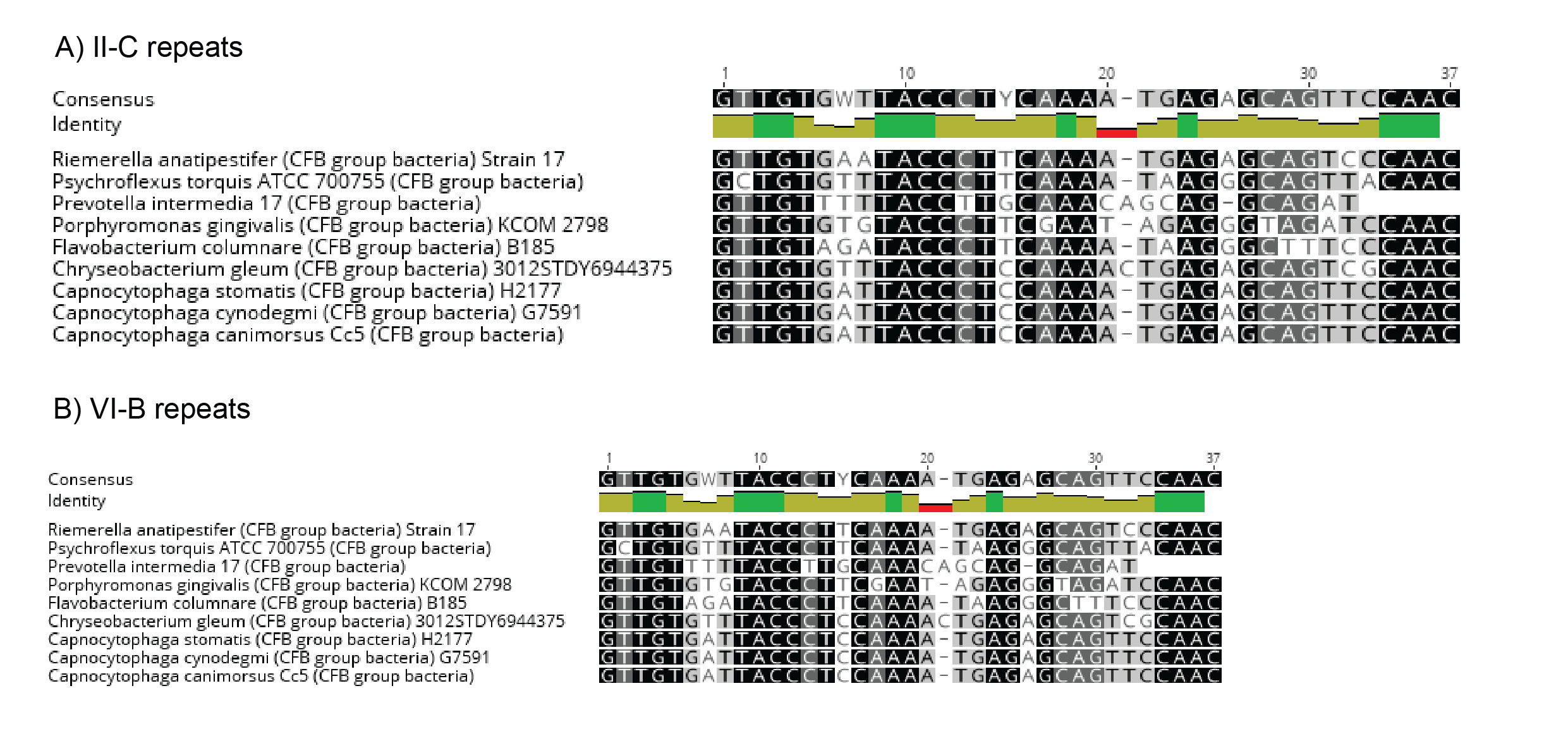

Supplement: FIG S5 [file mBio.03338-20-sf005.tif]
