# Supplementary material for: Cooperation between Different CRISPR-Cas Types Enables Adaptation in an RNA-Targeting System
Source: mBio. 2021 Mar 30;12(2):e03338-20. doi: 10.1128/mBio.03338-20 (PMC8092290; doi:10.1128/mBio.03338-20)
Supplement: FIG S6 [file mBio.03338-20-sf006.pdf]

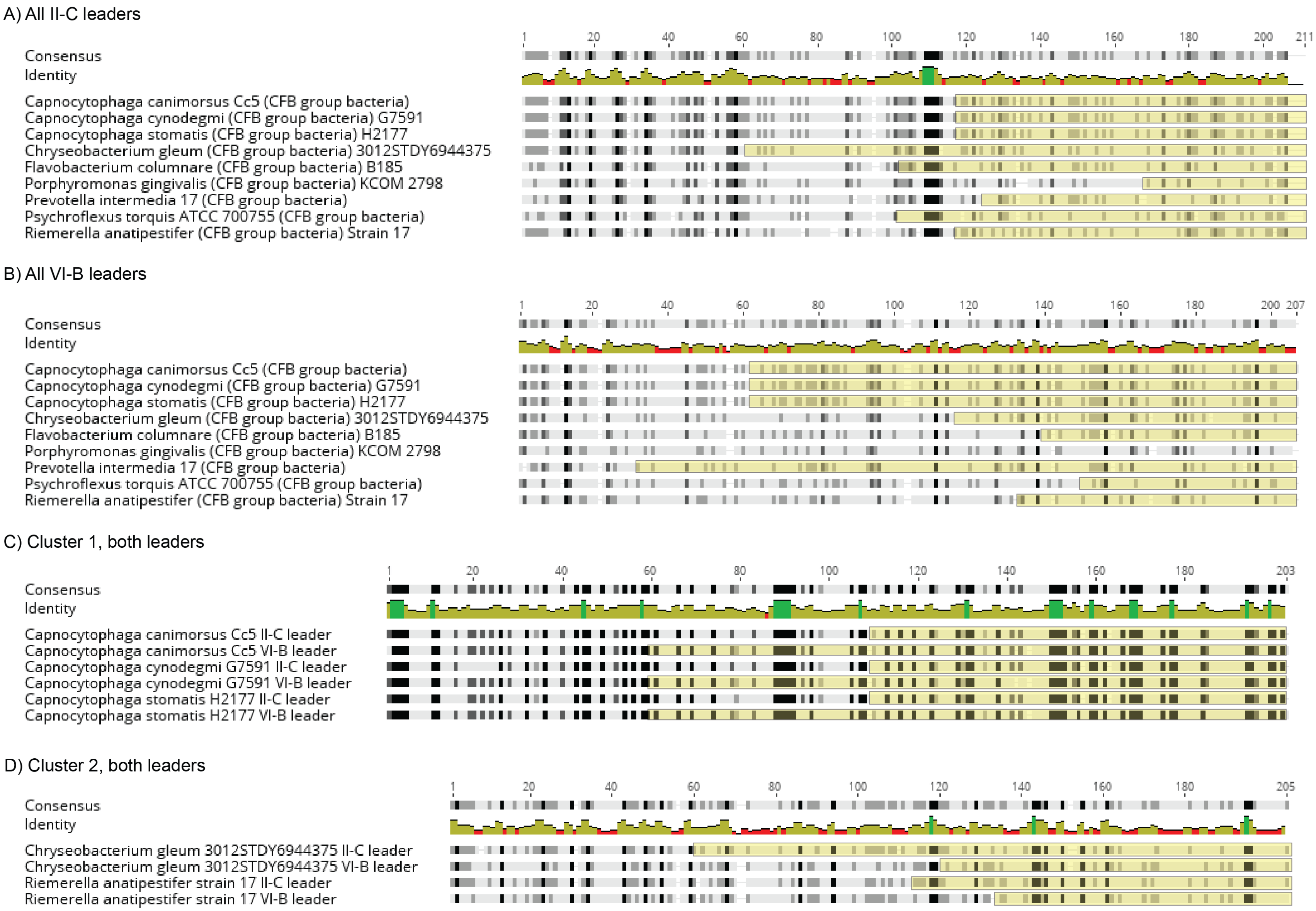
A) All II-C leaders
1
20
40
60
80
100
120
140
160
180
200
211
Consensus
Identity
Capnocytophaga canimorsus Cc5 (CFB group bacteria)
Capnocytophaga cynodegmi (CFB group bacteria) G7591
Capnocytophaga stomatis (CFB group bacteria) H2177
Chryseobacterium gleum (CFB group bacteria) 3012STDY6944375
Flavobacterium columnare (CFB group bacteria) B185
Porphyromonas gingivalis (CFB group bacteria) KCOM 2798
Prevotella intermedia 17 (CFB group bacteria)
Psychroflexus torquis ATCC 700755 (CFB group bacteria)
Riemerella anatipestifer (CFB group bacteria) Strain 17
B) All VI-B leaders
1
20
40
60
80
100
120
140
160
180
200
207
Consensus
Identity
Capnocytophaga canimorsus Cc5 (CFB group bacteria)
Capnocytophaga cynodegmi (CFB group bacteria) G7591
Capnocytophaga stomatis (CFB group bacteria) H2177
Chryseobacterium gleum (CFB group bacteria) 3012STDY6944375
Flavobacterium columnare (CFB group bacteria) B185
Porphyromonas gingivalis (CFB group bacteria) KCOM 2798
Prevotella intermedia 17 (CFB group bacteria)
Psychroflexus torquis ATCC 700755 (CFB group bacteria)
Riemerella anatipestifer (CFB group bacteria) Strain 17
C) Cluster 1, both leaders
1
20
40
60
80
100
120
140
160
180
203
Consensus
Identity
Capnocytophaga canimorsus Cc5 II-C leader
Capnocytophaga canimorsus Cc5 VI-B leader
Capnocytophaga cynodegmi G7591 II-C leader
Capnocytophaga cynodegmi G7591 VI-B leader
Capnocytophaga stomatis H2177 II-C leader
Capnocytophaga stomatis H2177 VI-B leader
D) Cluster 2, both leaders
1
20
40
60
80
100
120
140
160
180
205
Consensus
Identity
Chryseobacterium gleum 3012STDY6944375 II-C leader
Chryseobacterium gleum 3012STDY6944375 VI-B leader
Riemerella anatipestifer strain 17 II-C leader
Riemerella anatipestifer strain 17 VI-B leader

E) Cluster 3, both leaders

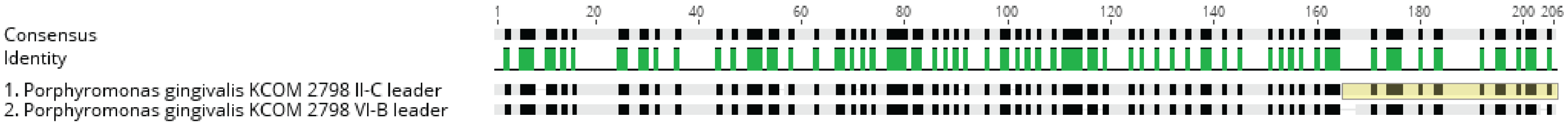

F) Cluster 4, both leaders

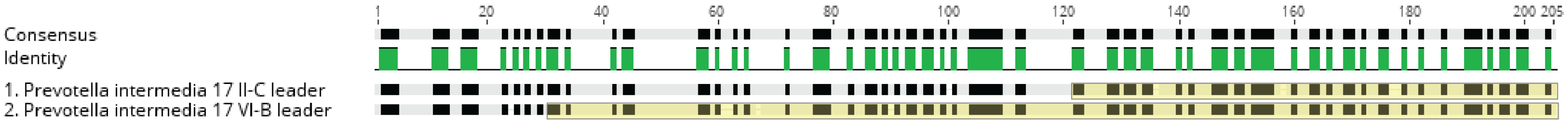

G) Cluster 5, both leaders

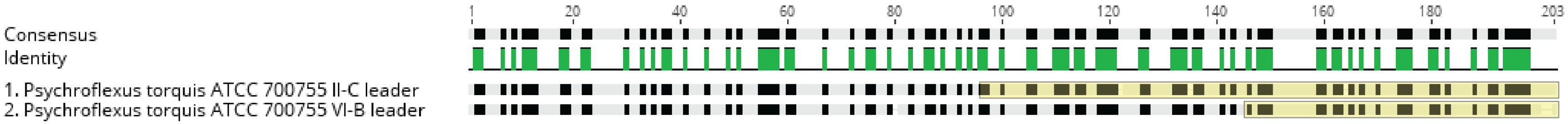

H) Cluster 6, both leaders

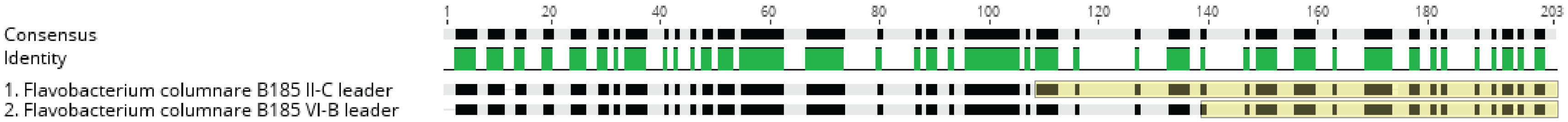

I) Cas1 tree & clusters

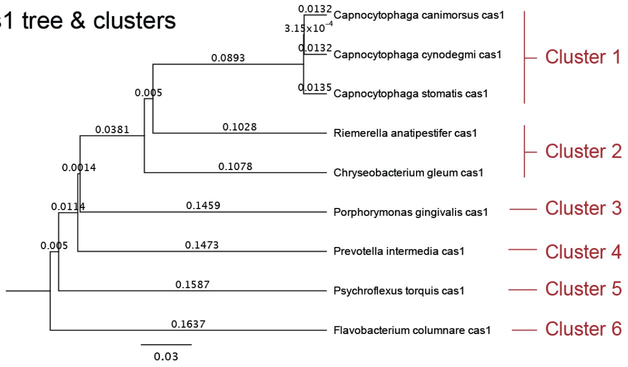

J) Similarity of leaders in clusters

Cluster	II-C & VI-B leader similarity
1. Capnocytophaga (canimorsus, cynodegmi, stomatis)	58.6 %
2. Chryseobacterium gleum, Riemerella anatipestifer	38.7 %
3. Porphyromonas gingivalis	40.3 %
4. Prevotella intermedia	42.3 %
5. Psychroflexus torquis	41.9 %
6. Flavobacterium columnare	47.8 %
